# Three-dimensional morphodynamic simulations of macropinocytic cups

**DOI:** 10.1016/j.isci.2021.103087

**Published:** 2021-10-01

**Authors:** Nen Saito, Satoshi Sawai

**Affiliations:** 1 Exploratory Research Center on Life and Living Systems, National Institutes of Natural Sciences, 5-1 Higashiyama, Myodaiji-cho, Okazaki, Aichi 444-8787, Japan; 2 Department of Basic Science, University of Tokyo, Meguro-ku, Tokyo 153-8902, Japan; 3 Research Center for Complex Systems Biology, Graduate School of Arts and Sciences, University of Tokyo, Meguro-ku, Tokyo 153-8902, Japan; 4 Department of Biological Sciences, Graduate School of Science, University of Tokyo, Bunkyo-ku, Tokyo 113-0033, Japan

**Keywords:** Membrane architecture, Cell biology, Computer modeling

## Abstract

Macropinocytosis refers to the non-specific uptake of extracellular fluid, which plays ubiquitous roles in cell growth, immune surveillance, and virus entry. Despite its widespread occurrence, it remains unclear how its initial cup-shaped plasma membrane extensions form without any external solid support, as opposed to the process of particle uptake during phagocytosis. Here, by developing a computational framework that describes the coupling between the bistable reaction-diffusion processes of active signaling patches and membrane deformation, we demonstrated that the protrusive force localized to the edge of the patches can give rise to a self-enclosing cup structure, without further assumptions of local bending or contraction. Efficient uptake requires a balance among the patch size, magnitude of protrusive force, and cortical tension. Furthermore, our model exhibits cyclic cup formation, coexistence of multiple cups, and cup-splitting, indicating that these complex morphologies self-organize via a common mutually-dependent process of reaction-diffusion and membrane deformation.

## Highlights

• A mathematical model of macropinocytosis cup formation and closure is proposed
• A self-enclosing cup emerges from reaction-diffusion pattern on a deformable membrane
• Cup-like membrane deformation can arise without curvature-inducing molecules
• Cup morphology variations explain the conditions and efficiency of fluid uptake

## Graphical abstract

**Figure:**
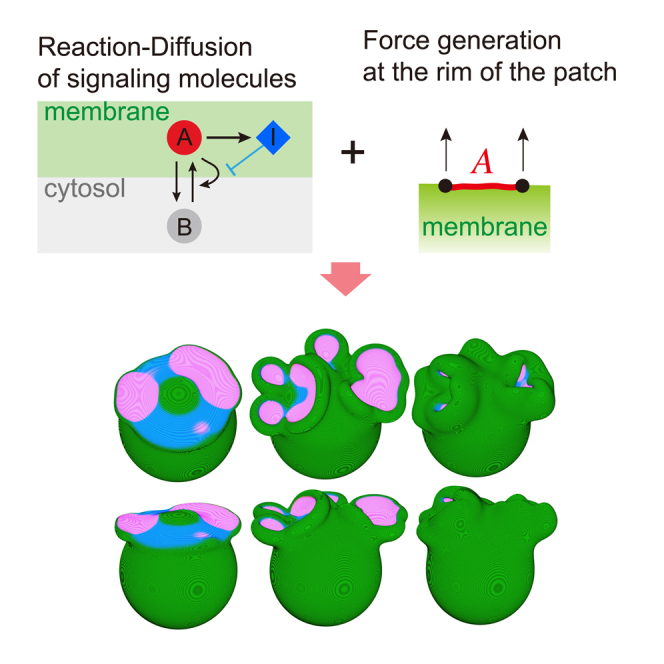

## Introduction

**Figure 1:**
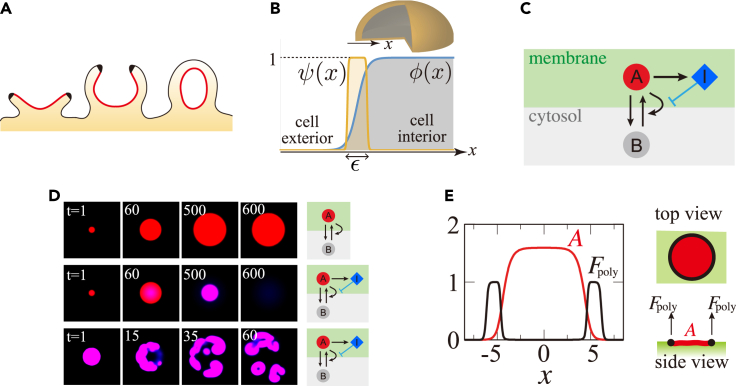
Formation of the macropinocytic cup and model schematics (A) Time sequence of macropinocytic cup formation (left to right). A micrometer-scale membrane domain; “active patch” (red) enriched in small GTPases and phosphoinositides grows and expands in the plasma membrane. The Scar/WAVE complex is localized at the edge of a patch (black) (Veltman et al., 2016). (B) Phase field φ defines the state of position *x* in space; occupied (φ=1) or vacant (φ=0). An auxiliary variable ψ is introduced to delineate the border (ψ=1) i.e., the plasma membrane and the rest of the space (ψ=0). (C) Schematic diagram of the model reaction.(A and B) are the active and inactive forms of an active patch factor, respectively. I is a factor that suppresses the positive feedback amplification ofA at the membrane. (D) The patch dynamics on a static flat surface. A static spot pattern forms when the inhibitor is absent (upper panel; $D_{a}=0.1$, α=1.0,$a_{\text{t}}=2.6$). For the presence of the inhibitor, the spot disappears with a finite lifetime (middle panel; $D_{a}=0.1$, $D_{i}=0.01$, α=1.0,$a_{\text{t}}=2.6$, $k_{1}=k_{2}=2.0\times 10^{-4}$) or continues to split and expand (bottom panel; $D_{a}=0.085$, $D_{i}=0.11$, α=50.0,$a_{\text{t}}=1.985$, $k_{1}=0.088,\;k_{2}=0.54$). (E) The spatial profile of protruding force *F*_poly_ (Equation 7) is determined by the distribution of A. A representative data for a 2D-planar membrane (K = 0.005, $K^{\prime }$ = 0.25 and $n_{h}=3$).

Macropinocytosis is an evolutionarily conserved actin-dependent endocytic process (King and Kay, 2019), in which the extracellular fluid is taken up by internalization of micrometer-scale cup-shaped membrane ruffles (Figure 1A). A wide range of cell types exhibit macropinocytosis either in a constitutive manner or under growth or other stimulating signals. Macropinocytosis is employed for nutrient uptake in *Dictyostelium* (Hacker et al., 1997) and certain cancer cells (Commisso et al., 2013; Kamphorst et al., 2015). In immune cells, macropinocytosis plays a role in surveying foreign antigens (Norbury, 2006; Yoshida et al., 2009; BoseDasgupta and Pieters, 2014; Condon et al., 2018). In neurons, macropinocytosis is employed to regulate neurite outgrowth (Kabayama et al., 2011). Understanding the basis of these processes is of biomedical importance because of its link to tumor growth (Commisso et al., 2013; Kamphorst et al., 2015), virus entry (Mercer and Helenius, 2009), and spread of prions related to neurodegenerative disease (Yerbury, 2016). Despite the wide occurrence of these phenomena, the basic question regarding the nature of the membrane deformation remains unanswered. Large-scale cup formation involves complex spatiotemporal regulation of signaling molecules and the cytoskeletal machinery. Unlike the better-studied clathrin-coated pits, where membrane invagination of ∼100 nm diameter is formed by the clathrin assembly, macropinosomes have no apparent coat structures and their size varies between 0.2 and 5 μm in diameter (Hewlett et al., 1994; Swanson, 2008; Yoshida et al., 2009). Furthermore, in contrast to phagocytic cups, which extend along the extracellular particles (Herant et al., 2006; Richards and Endres, 2017) (e.g., other cells to be engulfed for phagocytes, and pathogens for immune cells), there is no such support to guide macropinocytic cups externally. These morphological and dynamical features that are distinct from other endocytic processes indicate a mechanism unique to macropinocytosis that remains to be elucidated.

While there are morphological variations in the cup forming dynamics across cell types and species, there is an array of conserved signaling molecules that take part in the process (King and Kay, 2019). A well-studied case is that in amoeba *Dictyostelium*. Here, the initial stage of cup formation is identifiable by the formation and expansion of a patch-like localization of signaling molecules in the plasma membrane, which consists of phosphatidylinositol (3,4,5) trisphosphate (PIP3) and the active form of small GTPases such as Ras, Rap, and Rac surrounded by an edge region enriched in F-actin, Arp2/3, and the Scar/WAVE complex that drives membrane protrusions (Swanson, 2008; Buckley and King, 2017). Similar appearance of cell surface ruffles enriched in PI3K and PIP3 are also known in fibroblasts and macrophages (Swanson and Yoshida, 2019). For brevity, we hereafter refer to this membrane domain as the ‘active patch’. The relative positioning of these factors remains fixed as the patches grow in size (Figure 1A, left). The edges of the active patches protrude outward up to several micrometers, thus forming the rim of a cup, which then curves inward to ingest extracellular fluid (Figure 1A, middle). The resulting cup closes by membrane fusion to form a macropinosome (Figure 1A, right), which further matures and fuses with lysosomes for degradation of incorporated extracellular solutes (Buckley and King, 2017). The active patch is thought to self-organize through a combination of autocatalytic activation of Ras and PIP3 production and their diffusion (Weiner et al., 2007; Taniguchi et al., 2013; Bernitt et al., 2017; Flemming et al., 2020). When observed in the ventral membrane along the plane of contacted substrate, active patches appear as traveling spots and waves, a hallmark of reaction-diffusion-mediated pattern formation (Bretschneider et al., 2004; Weiner et al., 2007; Itoh and Hasegawa, 2013; Taniguchi et al., 2013; Gerhardt et al., 2014). Although these active patches appear to act as a prepattern or ‘template’ for macropinocytic cups (Veltman et al., 2016), little is known how these materialize into the formation of the cup itself.

In recent years, progress in theoretical and computational approaches has allowed the dynamical properties of cellular and sub-cellular scale membrane deformation, such as amoeboid motion and filopodia formation. Common to these modeling approaches is a mathematical formulation that describes the underlying regulatory kinetics together with a moving boundary. This physico-chemical coupling makes the problem unique and challenging because the very nature of highly deformable boundaries requires elaborate techniques to solve the interface physics that are often computationally laborious and expensive. Many studies have focused on cases that can be approximated in one- and two-dimensional space, including but not limited to the formation of filopodia during axonal elongation (Najem and Grant, 2013), pseudopodium in ameboid migration (Moure and Gomez, 2016), and lamellipodia of fish keratocytes (Shao et al., 2010; Shao et al., 2012; Lee, 2018), while relatively few attempts have been made for three-dimensional dynamics (Tjhung et al., 2015; Campbell et al., 2018; Rueda-Contreras et al., 2018; Cao et al., 2019). Models of 2-D dynamics by the active patches constrained to the ventral (Taniguchi et al., 2013) or dorsal side (Bernitt et al., 2017) of the plasma membrane were analyzed. Given the topological changes associated with cup formation and closure of the membrane, understanding the full nature of membrane deformation in macropinocytosis poses a challenge that requires full 3-dimensional modeling. In this paper, we propose and analyze a minimalistic 3-D model to address the relationship between the self-organizing active patches and the geometry of macropinocytic cup formation and closure. Our results indicate that a relatively simple rule of self-organization coupled with membrane protrusion can explain the entire sequence of the dynamics starting from patch expansion, cup formation, and cup closure without further need for specialized machineries to regulate local curvature.

### Model

We adopt a modeling strategy that combines two elementary processes: (1) deformation of the membrane and (2) the reaction-diffusion process of signaling molecules on the deformable membrane. To describe the membrane, we employed the phase-field method, which allows the simulation of interfaces with complex geometries such as growing crystals (Karma and Rappel, 1998; Beckermann et al., 1999), vesicle coarsening or fission (Lowengrub et al., 2009), the overall shape of migratory cells (Shao et al., 2010; Najem and Grant, 2013; Taniguchi et al., 2013; Flemming et al., 2020) and the signal localization on the cell membrane (Levine and Rappel, 2005). The phase-field approach allows the computation of cellular membrane deformation on the order of micrometers on a spatial scale and seconds to minutes in timescales, which is in contrast to nanometer-scale models that describe microsecond order phenomena (Sadeghi and Noé, 2020). Here, an abstract field variable φ is introduced to describe the cell interior region φ=1and the exterior region φ=0 (Figure 1B). φ is assumed to be continuous and varies sharply at the interface with finite width characterized by a small parameter ε. Following previous studies (Shao et al., 2010; Taniguchi et al., 2013; Camley et al., 2017), we adopt the following equations (see METHOD DETAILS for derivation):

(Equation 1) $$\tau \frac{\partial \varphi }{\partial t}=\eta \left(\nabla^{2}\varphi -\frac{G^{\prime }\left(\varphi \right)}{\varepsilon^{2}}\right)-M_{V}\left(V-V_{0}\right)\left|\nabla \varphi \right|+F_{\text{poly}}\left|\nabla \varphi \right|,$$

where$G^{\prime }=16\varphi \left(1-\varphi \right)\left(1-2\varphi \right)$ and V=∫φdr. The first term on the right-hand side represents the curvature-driven force associated with surface tension η. The second term imposes a constraint on the cell volume to $V_{0}=4\pi R_{0}^{3}/3$ where $R_{0}$ is the cell radius and $M_{V}$ is a constraint parameter. The third term describes the force normal to the membrane surface by dendritic actin polymerization. The magnitude of the force $F_{\text{poly}}$ is assumed to be a function of the local concentrations of signaling molecules, as described below. We note that the first term serves to minimize the total surface area, however, there is no area conservation constraint in the present scheme. Experimental measurements in *Dictyostelium* cells have shown that the cell surface area is not preserved but constantly fluctuating (Traynor and Kay, 2007) likely by an endocytic process other than macropinocytosis (Aguado-Velasco and Bretscher, 1999).

For the time development of the active patch signaling, let us assume a scheme where it takes two forms: the active form ‘*A*’ on the plasma membrane and the inactive cytosolic form ‘*B*’. We consider an interconversion A⇆B (Figure 1C) while the total number of ‘*A*’ and ‘*B*’ is fixed to$\;A_{\text{t}}$. The conversion from ‘A’ to ‘B’ is assumed to occur at a constant rate, whereas that of ‘B’ to ‘A’ is facilitated in an autocatalytic manner. The scheme gives rise to bi-stability, where A takes two states: zero and a finite positive value. When *A* is locally perturbed from A=0, a domain with elevating *A* spreads in space and eventually stops owing to the depletion of *B*, thereby creating a stable spot pattern (Figure 1D upper panel; Video S1A). Additionally, we shall further introduce a factor ‘*I*’ that is localized on the membrane and inhibits the conversion of ‘*B*’ to ‘*A*’ so that the active patch has a finite lifetime (Figure 1D middle panel; Video S1B) or can propagate (Figure 1D bottom panel; Video S1C). The above basic reactions are expressed in the following dimensionless reaction-diffusion equations (see METHOD DETAILS for derivation):

(Equation 2) $$\frac{\partial A}{\partial t}=\frac{A^{2}B}{1+A^{2}/\alpha^{2}}\frac{1}{1+I}-A+D_{A}\nabla^{2}A\;$$

(Equation 3) $$\frac{\partial I}{\partial t}=k_{1}A^{2}-k_{2}I+D_{I}\nabla^{2}I$$

where $D_{A}$ and $D_{I}$ are the diffusion constants of ‘A’ and ‘I’ molecules, respectively. The parameter α dictates the half-saturation concentration of the Hill function in the autocatalytic reaction B→A. The second equation assumes a negative feedback that produces inhibitor *I* at rate $k_{1}A^{2}$ and degrades at a constant rate $k_{2}$. When diffusion of ‘B’ molecule is sufficiently fast, $B=A_{\text{t}}/S-\langle A\rangle$, where *S* is the cell surface area, and $<A>$ is the total of A divided by S. Note that ‘A’ and ‘B’ can also be membrane-bound factors as long as diffusion of ‘B*’* is sufficiently fast compared to that of *‘*A’. For I=0 and $k_{1}=0$, the reaction-diffusion equations are reduced to the well-studied wave pinning model of cell polarization (Mori et al., 2008; Diegmiller et al., 2018).

Video S1. Typical patch dynamics of the biochemical reaction model, related to Figure 1 Timelapse images for the representative simulation. The parameter-set for (A), (B) and (C) corresponds to that for the top, middle and bottom panel in Figure 1D

To define the spatial coordinates occupied by the plasma membrane, we introduce an auxiliary phase-field $\psi ={\left(1+e^{-\beta \left(\varphi \left(1-\varphi \right)-\theta \right)}\right)}^{-1}$ which specifies the interface between the cell exterior (φ=0) and interior (φ=1) regions. ψ takes a constant value ψ=1 at the cell membrane and ψ=0 elsewhere (Figure 1B). Here, β takes a sufficiently large value to render the interface between the inside and outside of the membrane sharp, which ensures that the membrane-bound factors do not diffuse out of the membrane (Kockelkoren et al., 2003). θ is set so that the ψ is non-zero at the interface of φ with thickness ε. The unique aspect of the present approach is the introduction of this deformable auxiliary field ψ thereby allowing Equations (2) and (3) to be solved numerically at the interface only. In contrast, previous 2D models (Shao et al., 2010; Shao et al., 2012; Taniguchi et al., 2013) made distinctions only between occupied (cell; φ=1) and vacant (no-cell;φ=0) regions and assumed reactions that take place throughout the occupied space. Using ψ, the surface area is obtained by calculating $S=\int \psi /\varepsilon \;{\text{dr}}^{3}$, and the reaction-diffusion dynamics on the membrane follow

(Equation 4) $$\frac{\partial }{\partial t}\psi A=-\nabla \cdot \left(\psi Av\right)+\psi \left[\frac{A^{2}B}{1+A^{2}/\alpha^{2}}\frac{1}{1+I}-A\right]+D_{A}\nabla \left(\psi \nabla A\right)$$

(Equation 5) $$\frac{\partial }{\partial t}\psi I=-\nabla \cdot \left(\psi Iv\right)+\psi \left[k_{1}A^{2}-k_{2}I\right]+D_{I}\nabla \left(\psi \nabla I\right)$$

where the first terms on the right-hand side are the advection terms, and v is given by

(Equation 6) $$v=-\frac{1}{\tau }\left[\frac{\eta \left(\nabla^{2}\varphi -\frac{G^{\prime }\left(\varphi \right)}{\varepsilon^{2}}\right)}{\left|\nabla \varphi \right|}-M_{V}\left(V-V_{0}\right)+F_{\text{poly}}\right]\frac{\nabla \varphi }{\left|\nabla \varphi \right|}.$$

with the unit vector −∇φ/|∇φ| that defines the direction normal to the membrane.

The protrusive actin filaments are concentrated at the edge of the active patch (Veltman et al., 2016; Buckley and King, 2017). In the model, we assume that protrusion is promoted at a certain range of *A* as illustrated in Figure 1E. To implement this, the actin-dependent force normal to the membrane is described by the force term in the following form in Equations (1) and (6) according to:

(Equation 7) $$F_{\text{poly}}\left(A\left(r\right)\right)=F\frac{{\left(A/K\right)}^{n_{h}}}{1+{\left(A/K\right)}^{n_{h}}}\frac{1}{1+{\left(A^{2}/K^{\prime }\right)}^{n_{h}}}$$

so that $F_{\text{poly}}\left(A\right)\;∼\;F$ for $K≲A≲\sqrt{K^{\prime }}$. Instead of this minimalistic formulation, the localization at the edge of the domain can be incorporated by additional reaction-diffusion equations (Wigbers et al., 2020). The parameters of the model are listed on the table in the STAR Methods section.

## Results

### Mutual dependency between patch dynamics and deformation drives cup formation and closure

**Figure 2:**
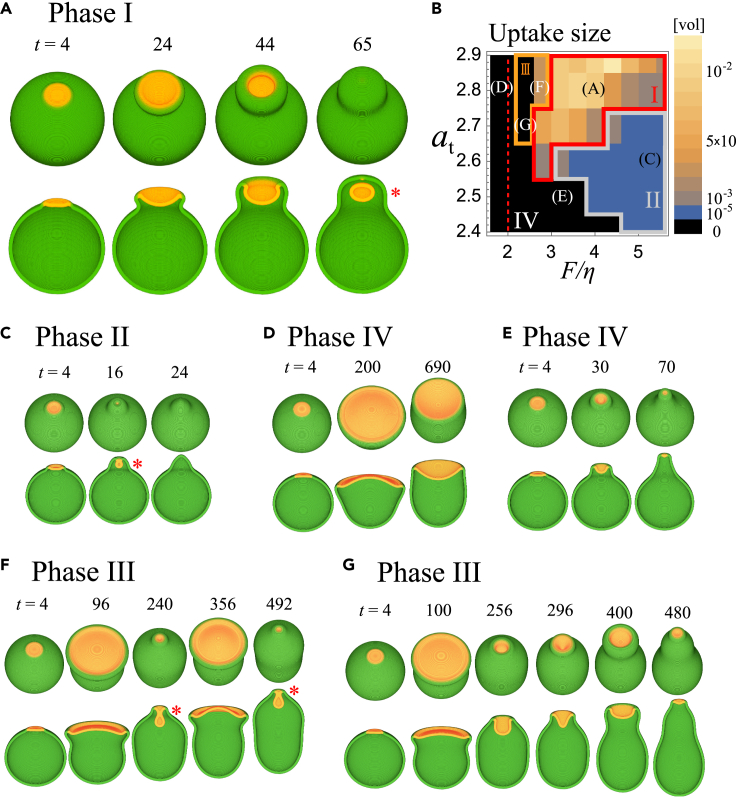
Membrane protrusion at the edge of an active patch is sufficient for the formation of the basic cup-like structure and its closure (A) A representative time course of the numerical simulations (F/η=4.0, $a_{t}=2.8$). The active patch (red; Aψ>0) and the membrane (green; ψ>0) shown as merged RGB images; birds-eye view (upper panels) and as cross-sections along the median plane (lower panels). Asterisks indicate cup closure. (B) Phase diagram of the cup dynamics. Color bars indicate the volume of enclosure normalized by the cell size (blue to yellow). The cut-off volume for successful cup closure was set to $<\;10^{-5}$ (black). Averages of six independent simulation runs (three of each for $r_{\text{init}}=1.0\;\text{μm}$and 1.5μm) are shown. Phase I and II: enclosure in all or part of the six trials, respectively. Phase III: repetitive cup formation. Phase IV: cup closure failed in all the simulations runs. The red dashed line is the estimated minimal force F/η=2.0required for protrusion. (C–G) Parameter sets in (A) and (C–G) are indicated in the diagram. (C–G) Representative time course for (C) F/η=5.2, $a_{\text{t}}=2.6$ (D) F/η=1.6, $a_{\text{t}}=2.8$, (E) F/η=3.2, $a_{\text{t}}=2.5$, (F) F/η=2.8, $a_{\text{t}}=2.8$, (G) F/η=2.4, $a_{\text{t}}=2.7$. Other parameters: τ=10, $D_{a}=0.1$, α=1.0, ε=0.8, $M_{V}=5.0$, *β* = 100.0, *θ*= 0.105, η=0.5, K=0.005, $K^{\prime }=0.25$, $n_{\text{h}}=3$.

First, we demonstrate the overall time development of the 3-dimensional model in the absence of the inhibitor ‘I’ ($k_{1}=0$). For the initial condition, we chose a membrane sphere with A=I=0 except for a small circular region of radius $r_{\text{init}}$ where A at each grid takes random values between 0 and 5.0. Representative results are shown in Figure 2A (see also Video S2). Because of the bistability in *A*, the initial seed gave rise to a local active patch with high *A*, which began to invade the surrounding basal state of low *A* in the form of a propagating front (Figure 2A; *t* = 4 orange region). As the patch expanded, the membrane protruded at the patch periphery and formed a cup-shaped circular extension (Figure 2A; *t* = 4–24 orange and green border). After the patch grew to a certain size, the expansion slowed. At the same time, the protrusion formed an overhang, while the center of the patch curved slightly inward to form a cup (Figure 2A; *t* = 24–44). The rim of the cup shrunk and was annihilated as the membrane sealed itself to completely surround a large volume of extracellular space (Figure 2A; *t* = 65). The coordinated manner in which a circular ruffle extended, shrunk, and closed showed a close parallel to the cup dynamics observed in *Dictyostelium* (Veltman et al., 2016). In addition, the marked accumulation of ‘A’ in the inner territory and its exclusion from the rim, which are in good agreement with the patterns of bona fide active patch marker$\;PIP_{3}$ and Ras-GTP (Hoeller et al., 2013; Veltman et al., 2014, Veltman et al., 2016; Williams et al., 2019a, 2019b). It should be noted that in the absence of membrane deformation and the inhibitor, a patch remains permanent once it reaches a certain size (Figure 1D upper panel). The patch shrinkage is thus a unique autonomous behavior that arises from the mutually defining nature of the patch patterning and membrane deformation. In *Dictyostelium*, the patch appearing in the ventral side of the plasma membrane interfaced with a flat substrate does not attenuate (Bretschneider et al., 2004; Taniguchi et al., 2013) which supports the notion that membrane deformation is essential for patch shrinkage. This contrasts to patch shrinkage in an earlier model of circular dorsal ruffle (Bernitt et al., 2017), which is described as a purely reaction-diffusion driven process.

Video S2. Cup formation and closure from a single founder patch, related to Figure 2 Timelapse images for the representative simulation result (F/η=4.0, $a_{\text{t}}=2.8$, $r_{\text{init}}=1.5\;\text{μm}$) shown in Figure 2A. Merged RGB images: (red, Aψ>0 (patch); green, ψ>0 (membrane))

**Figure 3:**
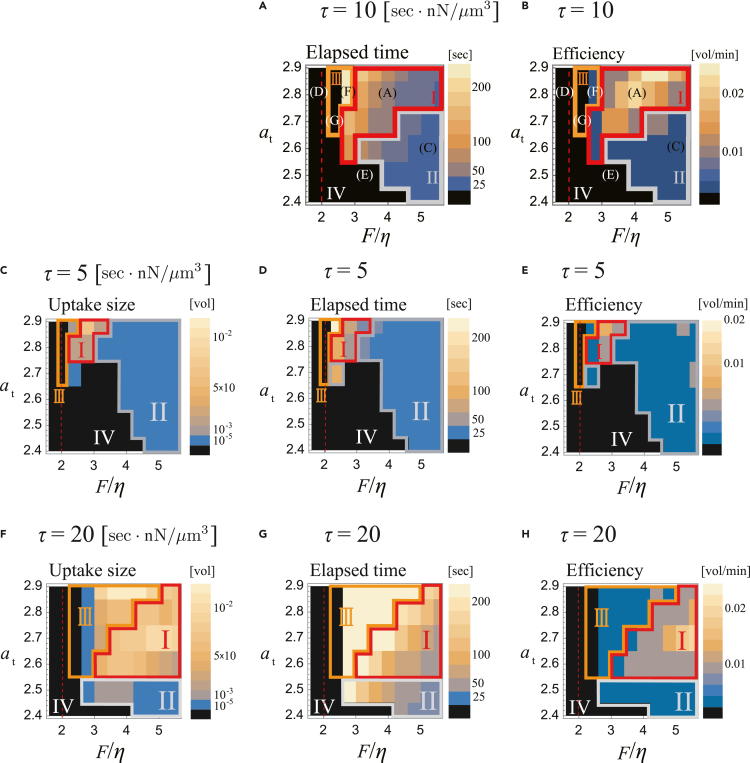
Deformation timescale acts critically for the intake volume and elapsed time for cup closure (A–H) Phase diagrams obtained from the quasi-3 dimensional space simulations for τ=10 (A, B), τ=5 (C–E) and τ=20 (F–H). The average fraction of the enclosed volume (C, F), the elapsed time from the patch initiation to cup closure (A, D, G), and the average fraction of the enclosed volume per minute (B, E, H). See Figure 2B for the intake volume for τ=10. In (A) and (B), parameter sets for Figures 2A and C–G are indicated in the respective letters. The averages are taken from six independent simulations (three of each $r_{\text{init}}=1.0\;\text{μm}$and 1.5μm). The enclosed volume smaller than $10^{-5}$ was scored as no uptake. For (A, B, D, E, G, H), averages are taken from successful enclosure. For phase III, only the last of the repeated enclosure was sampled for averaging. Other parameters are the same as in Figure 2.

Whether the enclosing cup successfully formed depended on the parameters and initial condition. We shall first highlight two key parameters critical for enclosure of a large volume: the membrane deformability and the patch size. The membrane deformability is described by the ratio between the force per unit area F and the surface tension η, i.e., F/η, while the patch size is controlled by the total amount of A and B per unit area $a_{\text{t}}\equiv A_{\text{t}}/4\pi R_{0}^{2}$. Figure 2B illustrates the phase diagram for cup closure, which was judged by quasi 3D simulations where dimensionality was reduced in an axis-symmetric coordinate for easier detection of morphology criteria and computation involving exhaustive parameter search (Figure S1A–S1H; see Methods). Black regions represent parameters that were unable to support closure; otherwise, color represents the enclosed volume relative to the cell volume $V_{0}$ (Figure 2B). The parameter space can be divided into four domains: Phase I – IV, based on the success rate of closure. Phase I includes the example shown in Figure 2A, where the parameters supported enclosure in all cases. Phase II consists of parameters in which cup closure depends on the initial conditions. Here, because of the small patch size, the cup and hence the enclosed extracellular volume was sometimes extremely small (Figure 2C). In Phase IV, cup closure failed in all simulation runs (Figures 2Dand 2E). In Phase III, cup formation repeated (Figures 2F and2G). All phase diagrams were obtained by averaging the results from two initial patch size $r_{\text{init}}$ (Figure S2A and S2B). A portrayal of the parameter space similar to Figure 2B was obtained based on the elapsed time between patch initiation and cup closure (Figure 3A), and ingestion efficiency (Figure 3B).

The requirements for successful cup formation and closure (Phase I) can be understood from the characteristic dynamics observed when cups fail to support large volume uptake. Phase IV consisted of two patterns of incomplete closure, depending on the value of F/η. When F/η was small, patches and cups persisted indefinitely without shrinking or closing (Figure 2D) for both high and low $a_{\text{t}}$. The behavior at low F/η was due to the lack of sufficient protrusive force for cup development. If we consider a cross-section of a protrusion with a width 2*R* (Figure S3), the force per unit length *F* exerted on the semicircular head of length $l_{h}=\pi R$ would be twice as large as the line tension η required to maintain the protrusion. Hence, the minimal force $F^{*}$ must obey $F^{*}/\eta =1/R=\;\text{π}/l_{h}$. Based on cortical tension of ∼0.7 nN/μm (Álvarez-González et al., 2015) and an estimate for protrusive force ∼6.5 to 9 nN/μm^2^ (protrusive force by a single microfilament 5 to 7 pN times the filament density (Abraham et al., 1999)), the condition F/η>1/R is satisfied for protrusion width of ≳ 0.2 μm. While $l_{h}$ in real cells has not been measured quantitatively, projections thinner than 0.2 μm would require larger F/η than the above estimate. The relative ease of imaging the cups under the conventional confocal microscope suggests that they are above the diffraction limit (>0.25 μm), which is within this force requirement. Because the spatial resolution of our numerical simulations were limited by the computational time, for systematic parameter studies, parameters K and $K^{\prime }$ in Equation (7) were chosen so that $l_{h}$ ∼ 1.5 μm (Figure 1D, black plateau; Figure S3), hence $F^{*}/\eta$ ∼ 2.0 μm^−1^ which is consistent with the boundary in the phase diagram (Figure 2B; red dashed line). Since the width of simulated protrusion is thicker than the above estimate (0.2 μm), 2–3 times weaker force value (0.8–2.8 $\text{pN}/\text{μ}{\text{m}}^{2}$) is sufficient to support protrusion (see Table in STAR METHOD). In contrast to the force constraints at small F/η, the characteristic behavior at high F/η (Figure 2E) was due to the lack of sufficient patch size at small $a_{\text{t}}$. Here, the resulting small cups gave rise to a high negative curvature, which in turn provided a strong restoring force in the inner territory that prevented the protrusion from curling inward. This resulted in a shmoo-like cell morphology (Figure 2E; *t* = 70), which eventually returned to the symmetric sphere as the patch disappeared. This patch attenuation was a distinct feature that arose due to the self-consistency requirement that the edge of the patch must define the point of protrusion and vice versa. If protrusions were to come close and coalesce due to high tension, the region surrounding it must also disappear. It should be noted that the same parameters support a persistent patch if it were not for deformation; thus, the coupling between the reaction-diffusion process and deformation is essential.

In Phase III, cup formation was repeated at the same site. In our simulations, there were two patterns of repetition, both of which occurred under conditions that allowed the formation of exceedingly large cups (Figures 2F and 2G). In the first example (Figure 2F; see also Video S3), the cup closed at its waist (Figure 2F; *t* = 240 s), while the remaining open half continued to expand at the edge (Figure 2F; *t* = 356). After the second closure (Figure 2F; *t* = 492), the rim disappeared and there was no more cup formation. The other pattern occurred for a slightly weaker force (Figure 2G; see also Video S4). Here, cup closure was stalled in the middle (Figure 2G; *t* = 296) as the patch continued to expand laterally before the next attempt at cup formation (Figure 2G; *t* = 400). Repetitive cup formation has been observed in the standard axenic strain of *Dictyostelium discoideum* and in an even more pronounced form in the null-mutant of RapGEF (*gflB*) (Inaba et al., 2017). While the distinction between these two behaviors predicted from our simulations is difficult to resolve experimentally, the markedly elongated cell shape (Figure 2F; *t* = 492 and Figure 2G; *t* = 480), and the lengthening of time required for enclosure (Figure 3A) were in accordance with those reported for the *gflB* mutant.

Video S3. Repetitive cup formation with cup closure at the waist, related to Figure 2 Timelapse images for the representative simulation result (F/η=2.8, $a_{\text{t}}=2.8$, $r_{\text{init}}=1.5\;\text{μm}$) shown in Figure 2F. Merged RGB images: (red, Aψ>0 (patch); green, ψ>0 (membrane))

Video S4. Repetitive cup formation with incomplete cup closure, related to Figure 2 Timelapse images for the representative simulation result (F/η=2.4, $a_{\text{t}}=2.7$, $r_{\text{init}}=1.5\;\text{μm}$) shown in Figure 2G. Merged RGB images (red, Aψ>0 (patch); green, ψ>0 (membrane))

Besides F/η and $a_{\text{t}}$, the other important parameter for enclosure of a large volume is the timescale of deformation τ. Its inverse 1/τ defines the membrane protrusion velocity per unit stress, and thus a larger τ denotes a slower deformation timescale relative to that of the biochemical reactions (i.e., patch expansion speed). The role of parameter τ is most evident in determining the size of each phases, which indicates that the timescale of the membrane deformation should be also in the appropriate range for the successful enclosure of the cup. In the examples shown above (τ=10sec nN/$\text{μ}{\text{m}}^{3}$), normal cup closure (Phase I) was predominantly observed, and Phase III was confined to a narrow domain between Phases I and IV (Figure 2B). For smaller τ (τ=5), Phase II that supports the formation of a small cup became dominant, and Phases I and III were both confined to narrow regions in the parameter space (Figures 3C–3E). In contrast, at large τ(=20), the phase I and III regions expanded (Figures 3F–3H). Overall, normal cup closure (Phase I) is realizable at moderate or largeτ, however for largeτ it comes at the cost of inviting repetitive dynamics that are often incomplete (Phase III) in addition to the overall process slowing down (Figure 3G), making the process less efficient (Figure 3H).

### Inhibitor and mass conservation determine the duration of patch and cup dynamics

Large cell-size cups are frequently observed in the axenic strain of *Dictyostelium* (Veltman et al., 2014; Veltman et al., 2016; Bloomfield et al., 2015); however, they do not exist indefinitely. The active patches are mostly transient and eventually vanish with a lifetime of a few minutes (Weiner et al., 2007; Gerisch et al., 2012; Itoh and Hasegawa, 2013; Taniguchi et al., 2013; Gerhardt et al., 2014). In our simulations, the active patches on their own have a finite lifetime when the presence of inhibitor I is non-negligible $(k_{1}\neq 0$). For $k_{1}=k_{2}=2.0\times 10^{-4}$, inhibitor I increases at a much slower timescale than the initial expansion of the active patch. Eventually, I becomes sufficiently high to suppress A, i.e., the active patch (Figure S4) when $a_{\text{t}}$ satisfies a certain condition (see METHOD DETAILS). In Phase III, the presence of the inhibitor repressed repetitive cup formation and abolished ruffle formation (Figures S4C and S4F), whereas no change was observed for Phases I and II.

**Figure 4:**
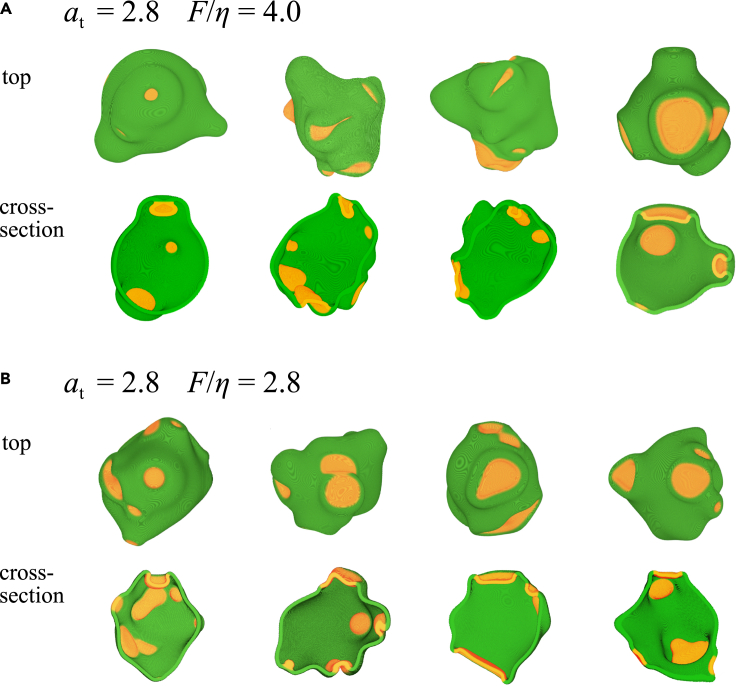
Complex cell morphologies result from multiple stochastic patch initiation (A and B) Representative snapshots from independent simulation runs with the same parameter sets as in Figure 2A and F (*I* = 0). Top overhead view (upper panels) and the midline cross-section (lower panels) with merged RGB images (Green: cell membrane (ψ>0). Red: active patches (*Aψ>* 0). Noise parameters: $\sigma =8.0,\;d=15.0,\;\lambda =3\times 10^{-5}$.

In addition to the inhibitor, the assumed mass conservation of the signaling molecule can also prevent the futile formation of excessively large cups. This effect becomes more evident when there are simultaneous and constitutive occurrences of active patches. Let us examine slightly complex situations where activation of A is allowed to occur at random positions $x_{\text{c}}$ at a rate θ per volume. The spatial profile of noise follows $N\left(x\right)=N_{0}\times \exp\left(-\frac{{\left|x-x_{\text{c}}\right|}^{2}}{2d^{2}}\right)$, where d is the initial nucleation size and $N_{0}$ is the noise intensity that follows an exponential distribution with average σ. Figure 4A shows representative snapshots from independent simulation runs (see also Video S5). A new active patch was nucleated before the existing cups closed, thus allowing multiple cups to coexist. Depending on the size and amplitude of the noise, some cups closed successfully, while others shrunk and vanished before they could close. Incomplete closure occurred even when the same parameter supported the closure of an isolated cup (Phase I). This can be explained by the effective reduction of $a_{\text{t}}$ available per cup. Due to continual cup formation and closure, the cell shape deviated markedly from the initial sphere and took a complex and processive morphology that highly resembled axenic strains of *Dictyostelium*. In the parameter regime that supported relatively large and slow cup closure (Phase 10.13039/501100000032III), these features became more exaggerated (Figure 4B). Multiple cups were frequently observed in *Dictyostelim* cells, and they either successfully closed to form endosomes or vanished without closing (Veltman et al., 2014).

Video S5. Multiple cup dynamics from stochastic patch initiation, related to Figure 4 Time-lapse images from a representative simulation with stochastic activation of A (F/η=4.0, $a_{\text{t}}=2.8$, $\sigma =8.0,\;d=15.0,\;\lambda =3\times 10^{-5}$). The other parameters are the same as seen in Figure 2A. Merged RGB images (red, Aψ>0 (patch); green, ψ>0 (membrane))

**Figure 5:**
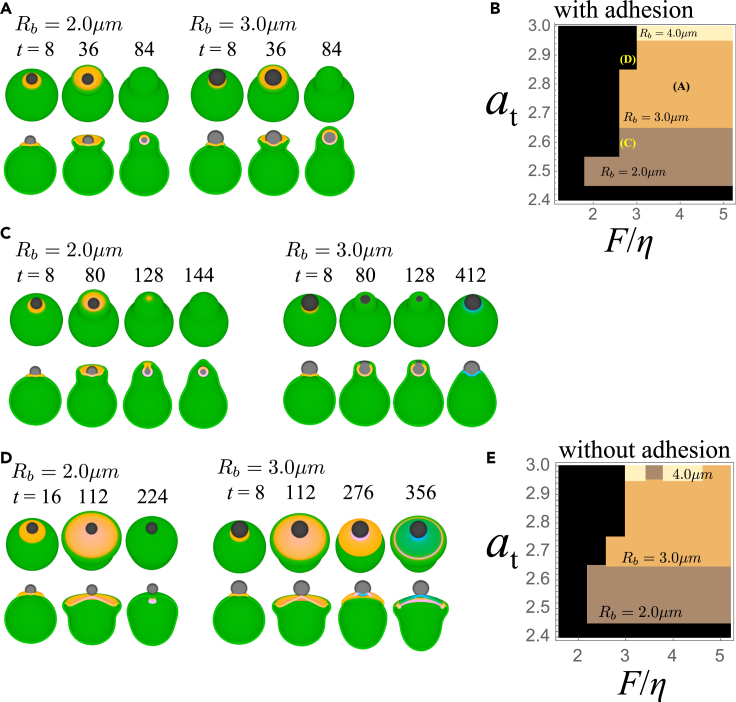
Engulfment of a solid particle (A) A representative time course $\left(F/\eta =4.0,a_{t}=2.8\right)$ for 2 μm (left panel) and 3 μm sphere (right panel). The active patch (red; Aψ>0), the membrane (green; ψ>0), the inhibitor (blue; Iψ>0) and the spherical particle (grey; *χ*> 0) shown as merged RGB images; birds-eye view (upper panels) and as cross-sections along the median plane (lower panels). (B) Phase diagram of complete engulfment (2.0, 3.0 and 4.0 micrometer radius spheres) in the presence of adhesion; $A_{\text{att}}=0.8$ and $A_{\text{rep}}=5.0$ (see also METHOD DETAILS). Black region: engulfment failed in all simulation runs. Parameter sets in (A) and (C and D) are indicated in the diagram. (C and D) Representative time course for (C) F/η=2.8, $a_{\text{t}}=2.6$ (D) F/η=2.8, $a_{\text{t}}=2.9$. (E) Phase diagram in the absence of adhesion; $A_{\text{att}}=0$ and $A_{\text{rep}}=5.0$. For all simulation, $k_{1}=k_{2}=$2.0$\times 10^{-4}$is used. Other parameters are the same as in Figure 2.

It is expected that the membrane cup and its closure should also be able to support the uptake of particles of micrometer size. To study this, we performed simulations with a spherical bead of either 2.0, 3.0, or 4.0 micrometer radius (see METHOD DETAILS). Representative results are shown in Figure 5A. When the cup formation occurred close to where a bead was attached (t = 8 and 36), the cup began to cover the bead, and eventually completely engulfed it (t = 84). The parameter region that supported complete engulfment is shown as a phase diagram (Figure 5B). Beads with radii of up to 4 μm can be taken up at high $a_{t}$ and large F/η. In contrast, only beads with a radius of 2 μm can be internalized at low $a_{t}$ (Figure 5C). At small F/η, the membrane protrusion took a long time due to weaker force, and hence the active patch vanished before beads could be internalized (Figure 5D). In the above simulations, adhesion between the cell membrane and the particle was included to stabilize the bead position close to the membrane. Particle uptake can still occur in the absence of adhesion though with less efficiency for large beads (Figure 5E).

### Excitability arises in the presence of a strong inhibitory signal and drives the cup-splitting dynamics

**Figure 6:**
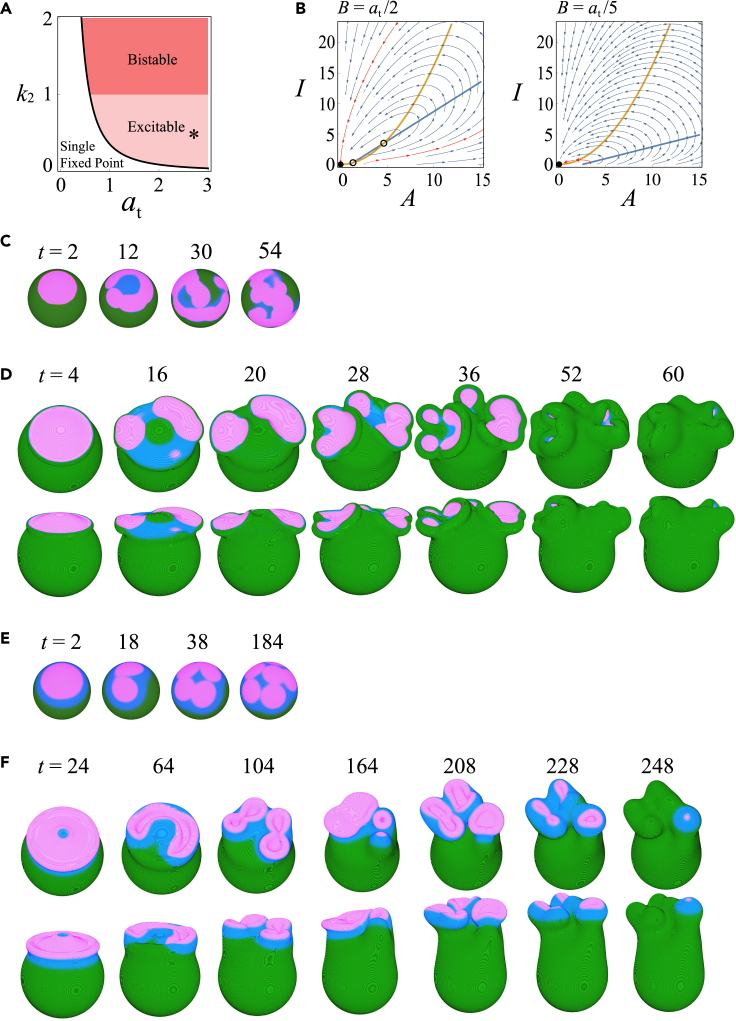
Presence of an inhibitor gives rise to cup-splitting dynamics (A) Phase diagram of the chemical reaction Equation (8) decoupled from deformation dynamics in the presence of inhibitor kinetics (Equation 3) for $k_{1}=0.088$. Depending on *k*_2_, the system is bistable ($k_{2}>1$) (red region) or excitable ($k_{2}<1)\;$(pink region). A single fixed point A=0 for $a_{t}<2\sqrt{k_{1}/k_{2}}$ (white region) and three fixed points A=0, and $A^{\pm }=\left(B\pm \sqrt{B^{2}-4k_{1}/k_{2}}\right)/\left(2k_{1}/k_{2}\right)\;$for $a_{t}>2\sqrt{k_{1}/k_{2}}$ (pink and red regions). A=0; stable. $A^{-}$; unstable. $A^{+}$; stable$\left(k_{2}>1\right)$ or unstable $\left(k_{2}<1\right)$. (B) Null-clines in the excitable regime for $B>2\sqrt{k_{1}/k_{2}}$ (left panel) and $B<2\sqrt{k_{1}/k_{2}}$ (right panel). Fixed points (Filled circle: stable. Open circle: unstable). Excitatory trajectories (red arrows) invoked by small perturbation to A=0. (C and D) Representative dynamics ($a_{\text{t}}=1.985$, $k_{1}=0.088$, $k_{2}=0.54,\;D_{a}=0.085$ and $D_{i}=0.11$) on a fixed spherical field (C) and deforming membrane (D) ($\tau =7.0,\;F=3.7,\;K_{1}=0.01,\;K_{2}=0.1$ and $n_{h}=5$). (E and F) Representative dynamics ($a_{\text{t}}=1.94$, $k_{1}=0.088$, $k_{2}=0.54,\;D_{a}=0.26$ and $D_{i}=0.87$) on a fixed spherical field (E) and deforming membrane (F) ($\tau =20.0,\;F=3.0,\;K=0.086,\;K^{\prime }=1.8$ and $n_{h}=3$). Other parameters are the same as in Figure 2.

The cup dynamics described above were monotonous, meaning that the initial active patch more or less dictated when and where a cup formed, and it grew due to bistability until it consumed all ‘B’. In *Dictyostelium*, however, cups are known to multiply or reduce in number by splitting and coalescence of existing cups (Veltman et al., 2016). In the present model, when the production of ‘A’ is no longer a saturating function (large α in Equation 2), the active patch (a region with a high A) can become out of phase with a high-*I* region. As a consequence, the region occupied by high I will trail behind a moving active patch and can disrupt it (Figure S5A and S5B). To study this behavior in detail, let us consider the case α→∞ so that Equation (2) now becomes

(Equation 8) $$\frac{\partial A}{\partial t}=\frac{A^{2}B}{1+I}-A+D_{A}\nabla^{2}A\;$$

which is the same equation introduced earlier as a part of a model for the patch dynamics in a circular dorsal ruffle (Bernitt et al., 2017). The key difference in the present model, apart from the incorporation of the membrane deformation, is that Equation (8) is coupled with Equation (3) with quadratic dependence on A which is essential for providing rich behavior as follows: The equation has three different parameter regimes–mono-stable, bi-stable, and excitable (Figure 6A, see also Figure S6A–S6C for finite α). In the excitable regime, null-cline analysis (Figure 6B, left panel) shows that for a small A (i.e., for $B>2\sqrt{k_{1}/k_{2}}$), a small perturbation from the fixed point A=0 gives rise to a large excitation of *A*. For a large A (i.e., for $B<2\sqrt{k_{1}/k_{2}}$: right panel in Figure 6A), excitability disappears and A falls immediately to the basal state even when strongly perturbed. Interestingly, this in turn brings the system back to an excitable state; hence, A is again easily perturbed and brought transiently to a high level. In other words, depending on A, i.e., the total size of the active patches, excitability is switched on and off in a sequential manner. This switching of excitability destabilizes the expanding front of the active patches (Figure 1D bottom panel, Figure S5A), similar to the splitting patches or waves observed on the ventral side of the plasma membrane (Bretschneider et al., 2004; Weiner et al., 2007; Itoh and Hasegawa, 2013; Taniguchi et al., 2013; Gerhardt et al., 2014).

When coupled to membrane deformation, however, a broad protrusive force profile in the region surrounded by a patch (Figure S5C and *D*; *t* = 8, 0 <*r*< 3) smoothed out the fragmented active patches before the daughter cups developed (Figure S5E and S5F). While this can be circumvented at small F, fragmented cups then failed to close due to lack of sufficient protrusion (Figure S5G). A recent CryoEM study of the ventral actin waves demonstrated that the form and alignment of actin filaments at the edge of the patch and those in the inner region are distinct, which suggests the presence of debranching factors that trail behind the expanding edge (Jasnin et al., 2019). This notion is consistent with the sharp localization of the Scar/WAVE complex at the edge of a patch (Veltman et al., 2016) and the depolymerization factor coronin at the rear of the edge (Bretschneider et al., 2009). To study such an effect in the model, let us modify the force term so that *I* not only suppresses amplification of *A* but also competitively inhibits force generation by A, so that

(Equation 9) $$F_{\text{poly}}\left(A\left(r\right),I\left(r\right)\right)=F\frac{{\left(A/K\right)}^{n_{h}}}{1+{\left(A/K\right)}^{n_{h}}}\frac{1}{1+{\left(I/K^{\prime }\right)}^{n_{h}}}$$

Note that the original form of $F_{\text{poly}}$ (Equation 7) is recovered when I is in the steady state, that is, $\dot{I}=0$, and $D_{I}$ is negligible. The periphery of the active patch is defined by a high A and low I*,* thus under Equation (9), the force profile is restricted to the edge (Figure S5H and S5I; *t* = 8). The protruding force in the inner territory only appeared later to surround the split patches (Figure S5H; *t* = 15). Accordingly, expanding active patches broke up repeatedly (Figures 6C–6F), while some of the daughter patches quickly merged with existing ones and gave rise to cup-shaped circular ruffles (Figure 6D; *t* = 20, 28, and Video S6). By ruffles, we mean that the rim of the cup was no longer smooth and circular but more undulated and complex in shape. Splitting of an active patch during ruffle formation causes fragmentation (Figure 6D; *t* = 36, and Figure 6F; *t* = 104). A notable difference from multiple cups occurring in the non-excitable regime (Figure 4) was that multiple patches and cups continued to emerge starting from a single founder. The sequence of events and their appearance, splitting followed by formation of cup-shaped ruffles (Figures 6D and 6F) are remarkably similar to how, in *Dictyostelium,* an active Rac-and F-actin-rich region expands together with membrane ruffles and then becomes fragmented into multiple macropinocytic cups (Veltman et al., 2016).

Video S6. Cup-splitting dynamics, related to Figure 6 Time-lapse images from representative simulations with I ($\tau =7.0,\;F=3.7,\;K=0.01,\;K^{\prime }=0.1$and $n_{h}=5$). The other parameters are the same as those in Figure 6*.* Merged RGB images (red, Aψ>0 (patch); blue, Iψ>0(inhibitor); green, ψ>0 (membrane))

## Discussion

The present work suggests an unexpectedly simple yet concerted mechanism that underlies the formation and closure of macropinocytic cups observed in *Dictyostelium*. A locally activated signaling patch, represented by a high A in the model, appears. The active patch then expands in a self-organized manner via autocatalytic transition of bistable nature from the state of low *A* to high *A*. From there, two key assumptions in the model dictate the fate of the patch and the resulting cup. (1) Limitation in the growth of an activated patch owing to the finite amount of signaling molecules (Equation (2)), that is, the sum of ‘A’ and ‘B’ molecules $A_{\text{t}}$ (or $a_{\text{t}}$ in the normalized form) is fixed. (2) Restriction of force protruding to the edge of an active patch (Equation (7) and (9)) (Bloomfield and Kay, 2016; Buckley and King, 2017). Owing to constraint (1), the patch first expands (Figure S7; *t* = *t*_1_), and then slows down as it reaches its size limit (Figure S7; *t* = *t*_2_). The edge continues to protrude and forces the patch area to expand. However, because *B* is no longer available, *A* at the patch boundary must be brought down to the low state. Thus, the position of the patch boundary (Figure S7; *t* = *t*_3_, black circle) was effectively displaced from the rim of the cup (Figure S7; *t* = *t*_3_, blue asterisk) toward the inner territory (Figure S7; *t* = *t*_3_). Because the protrusive force is generated at the patch boundary (2), the protrusion curves inward, forming an overhang (Figure S7; *t* = *t*_4_, *t*_5_) and continues to advance until they meet each other. The two basic properties (1) and (2) are sufficient for macropinocytic cup formation and closure, which was further demonstrated using a reduced model in the form of an ordinary differential equation (see Figure S8 and METHOD DETAILS) that describes an active patch boundary.

The membrane involution arises because of mutuality between the reaction-diffusion process and deformation dynamics in defining the position of the protruding rim. The present work thus brings to light a distinct mechanism of membrane invagination that contrasts with those driven by local curvature, for example, the formation of endocytic vesicles by clathrin (Lowengrub et al., 2016; Kaksonen and Roux, 2018) and BAR-domain-containing proteins (Frost et al., 2008; Lowengrub et al., 2016; Noguchi, 2016). Spontaneous curvature and the bending modulus were assumed to be negligible in the present model (Equation (1)). According to an earlier measurement, the bending modulus in *Dictyostelium* (Simson et al., 1998) is $K_{b}=400K_{b}T=1\text{∼}2\text{ pN}$⋅μm. For instance, a cylindrical membrane protrusion with radius r and length l, the bending energy $G_{b}\;$and the surface energy $G_{s}$ of the protrusion are $G_{b}\approx \pi K_{b}l/r$ and $G_{s}\approx 2\pi \eta rl$, and thus for r=1μm, $G_{b}/G_{s}\approx K_{b}/2\eta r^{2}\;∼10^{-3}.$ The bending energy for the micrometer scale deformation is thus two to three orders of magnitude smaller than the surface energy. Consistent with the estimate, incorporating the bending energy term (Shao et al., 2010, 2012) explicitly in the simulation had little effect on the results (Figure S9).

The high similarity between the range of complex morphology dynamics observed in the present simulations and those in *Dictyostelium* cells suggests that the kinetics adopted in the current model captured the essence of the underlying regulation and cell mechanics. The critical parameter that determines the occurrence of a patch and its size was $a_{\text{t}}$. Strong candidates for ‘A ’ and ‘B’ are the active and inactive forms of small GTPases such as Ras, Rap, and Rac, or their upstream and downstream signaling partners, such as PI3K, which are all found to be enriched in the active patch (Hoeller et al., 2013; Veltman et al., 2016; Miao et al., 2019). PI3K requires Ras binding for its activity (Funamoto et al., 2002; Sasaki et al., 2007); thus, variable A may represent Ras in a complex with PI3K or its product PIP3, and the variable B may be regarded as its inactive form. In fibroblasts, microinjection of active Ras protein induces macropinocytosis (Bar-Sagi and Feramisco, 1986). RasS mutations in *Dictyostelium* cells are known to inhibit macropinocytosis (Chubb et al., 2000; Hoeller et al., 2013). These perturbations can be understood from increasing or decreasing $a_{\text{t}}$ and, and hence, the size of the active patch. Owing to the nondimensionalization in Equation (2), the lowering of $a_{\text{t}}$ can also result from a decrease in the autocatalytic reaction B→A (see METHOD DETAILS). This analysis is in line with a recent suggestion based on the observation of smaller patches and macropinosomes in PI3K mutants (Veltman et al., 2016; Williams et al., 2019a, 2019b) and in a double mutant of Akt/PkbA and PkbR1 (Williams et al., 2019a, 2019b) likely that there is a positive feedback loop between PIP3 production and its downstream PKB in *Dictyostelium* (Williams et al., 2019a, 2019b).

Apart from the bistability, our model suggests that excitability arises when the self-amplification of A is less saturated (i.e., large α). In *Dictyostelium*, loss of Ras GTPase-accelerating protein (RasGAP) neurofibromin (NF1) causes the formation of oversized macropinosomes, increases fluid uptake, and facilitates cell growth in liquid media (Bloomfield et al., 2015). In our model, a decrease in RasGAP corresponds to a decrease in the rate of reactionA→B. Here too, due to parameter non-dimensionalization in Equation (2), this can be interpreted as an increase in $a_{\text{t}}$ or α (see METHOD DETAILS). Since elevation in αbrings the system to the excitable regime, attenuation of RasGAP makes it an ideal point of perturbation to enhance macropinocytosis, that is, an increase in the number of patches due to splitting, in addition to supporting a larger patch size. Both the expression of activated Ras in the wild-type cell (Williams et al., 2019a, 2019b) and Ras-GAP mutation (Bloomfield et al., 2015) are known to enhance fluid uptake. Our model assumes that inhibitor I weakens the autoregulatory amplification of A (the term ${\left(1+I\right)}^{-1}$ on the right-hand side of Equation 4)*.* From $\dot{I}=0$ at Equation (3), one can see that I imposes saturation in the production of A even at a high α, and thus has a similar effect to changing α. In addition, I critically affected patch duration as well as cup splitting (Figure 6D). For large $a_{\text{t}}$, the absence of inhibitor I caused cup formation to repeat at the same site due to incomplete closure of an oversized cup (Figure 2B; Phase III). Following the line of thought that ‘A’ may be regarded as an activated form of small GTPase, ‘I’ would be a factor that suppresses guanine nucleotide exchange factor (GEF)s. In line with the model behavior, a knockout of Ras/Rap GEF (GflB) does indeed repeat cup formation at the same site (Inaba et al., 2017).

Although the variables A and I are abstract and collective representations of regulatory factors, from the cell mechanics point of view, they must be closely linked to the nucleator Arp2/3 complex bound to the polymerizing actin (Bernitt et al., 2017), and a debranching factor such as coronin in complex with F-actin, respectively. Mass conservation of *A* + *B* in the model could hence be attributed to competition for a limited supply of actin or nucleating factors (Burke et al., 2014; Lomakin et al., 2015; Suarez and Kovar, 2016; Carlier and Shekhar, 2017; Antkowiak et al., 2019; Bleicher et al., 2020). In line with the global constraint, macropinocytic cup formation is known to compete with pseudopod formation (Veltman et al., 2014). The appearance of an active patch on one side of the plasma membrane excludes another patch from appearing at other locations (Helenius et al., 2018). As for variable I, the simulated profile (Figure S5B) is in line with that of coronin, which trails behind the traveling actin waves (Bretschneider et al., 2009). In addition to the role of I in inhibiting the amplification of A (Equation 2), our model assumed a nonlinear reaction term whereI increase by interacting with a duplex of A (Equation 3). We have also examined a version with a linear dependency on *A*, however, the parameter space that supported the excitability was drastically narrower (Figure S6D–S6F), and splitting was not observed. A possible interpretation of the nonlinear term would be to regard this as formation of a complex where actin filaments are cross-linked with coronin (De Hostos, 1999; Goode et al., 1999). In the active patch, coronin may mediate the switch in the orientation of the dendritic actin filaments from those facing the membrane to those that are parallel (Jasnin et al., 2019). Such a change would lead to a vanishing force in the direction normal to the membrane interface, consistent with our assumption that the force generated by A is competitively attenuated by I (Equation (9)). In some *Dictyostelium* mutants (Buckley et al., 2020), macropinocytic cups continue to fragment and give rise to new cups. This resembles our simulation with a small *F* (e.g., Figure S5G), whereas each fragmented patch fails to close in our simulation. Incorporating additional factors that modulate the position where the force is generated may cause such unceasing fragmentation and closure of the cups. With the inhibitor dynamics, because the patch annihilates by cup closure if not propagate indefinitely (Figure S5G), the net displacement of the cell centroid is close to zero. This contrasts with the directional cell movement driven by a propagating patch on a solid substrate (Honda et al., 2020).

In metazoan cells, morphology dynamics of macropinocytic cups are known to exhibit further complexities that are not addressed in the present study. In some cancer cells, the dorsal side of the plasma membrane is covered by a circular membrane ruffle associated with macropinocytosis (Itoh and Hasegawa, 2013; Bernitt et al., 2017). This so-called “circular dorsal ruffle” is initiated from F-actin-rich circular projections on the dorsal cell surface. Similar to the present simulations, the ring region expands and contracts, forming a cup-like structure. While restriction of the dynamics on the dorsal side can be explained by the presence of dorsal-ventral asymmetry in the parameter at Phase I (Figure 2A), multiple macropinosomes that form within a single cup (Bernitt et al., 2017) suggests an additional mechanism. Similarly, dendritic cells exhibit numerous multilayered membrane ruffles associated with macropinosomes (De Baey and Lanzavecchia, 2000; Chabaud et al., 2015). In macrophages, ruffling with a linear geometry near the cell edge fold back on itself to close the cup (Araki et al., 2000; Yoshida et al., 2009). Ruffles that accompany fine filopodial projections resembling a tent-pole are known to twist and circularize to form a cup (Condon et al., 2018). Further extension of the model, such as incorporating local changes in tension *η*, which likely depends on localized myosin I (Dai et al., 1999; Brzeska et al., 2016), constraints on the total surface area, an additional regulation that changes the speed of the vanishing vesicles, curvature dependency in the patch patterning (Honda et al., 2020), and other force profiles may help explain the complex morphological features. Simulations with higher spatial resolution and the bending modulus will also be important when studying the smaller ruffling. Furthermore, because the basic closure mechanism can work in pattern forming systems that satisfy a constant size spot formation, e.g., Turing-type patterning (Meinhardt, 1999) and noise-induced spot formation (Hecht et al., 2010), variations may also arise from reaction schemes that can give rise to more complex and chaotic patch patterns, where morphologies are far from symmetric and thus full three-dimensional analysis becomes more important. Future work should consider these possibilities when addressing the relationship between the distinct morphologies and the basic cup dynamics uncovered in this study.

### Limitations of the study

This study is limited by the spatial resolution employed when numerically integrating the partial differential equations. At present, with generally accessible GPU computation, the resolution dx = 0.1 μm with the width of the membrane ε = 0.8 μm is practical and was thus chosen. For this reason, detailed features of membrane deformation smaller than 1μm are outside the scope of this study. The present model also considers force normal to the membrane only. In real cells there may also be in-plane deformation arising from other processes. In such case, further complication such as chemical flow may arise and thus can potentially affect the pattern formation.

## STAR★Methods

### Key resources table

**Table:** 

REAGENT or RESOURCE	SOURCE	IDENTIFIER
**Deposited data**		

The cell surface tension parameter	(Álvarez-González et al., 2015)	https://doi.org/10.1016/j.bpj.2014.11.3478
The force magnitude parameter	(Abraham et al., 1999)	https://doi.org/10.1016/S0006-3495(99)77018-9
The bending modulus parameter	(Simson et al., 1998)	https://doi.org/10.1016/S0006-3495(98)77808-7

**Software and algorithms**		

Open GL	https://www.opengl.org/	version 2.1 INTEL-14.7.12
Simulation codes	–	https://github.com/nen6f/3D-simulations-of-macropinocytosis

### Resource availability

#### Lead contact

Further information and requests should be directed to and will be fulfilled by the Lead Contact, Nen Saito (n-saito@nibb.ac.jp).

#### Materials availability

The study did not generate any new materials.

### Method details

#### Phase field implementation

To simulate deformation of the plasma membrane a non-physical field φ is introduced. φ takes constant value (φ=1) at the cell interior region and (φ=0) at the exterior region, and varies sharply but smoothly in between. The width of the interface is characterized by the small parameter ε, which can be interpreted as the thickness of the membrane and the cortical layer. The time evolution of the interface between φ=0 and 1 is considered by the general interface advection equation

$$\begin{array}{c} \frac{\partial \varphi }{\partial t}+\vec{v}\cdot \nabla \varphi =0, \end{array}$$

where$\vec{v}$ is the velocity vector of the interface. We assume that the magnitude of the velocity is directly proportional to the force applied to the interface so that

$$\begin{array}{c} \vec{v}=\frac{\vec{F}+\vec{F_{c}}}{\tau }, \end{array}$$

where the coefficient τ has the unit of [N s/m^3^], $\vec{F_{c}}$ is curvature-driven force normal to the plasma membrane that results from surface tension γ. Given the curvature c, $\vec{F_{c}}=-\gamma c\vec{n}$. In the phase field, the unit vector normal to the interface and curvature are given by $\vec{n}=-\nabla \varphi /\left|\nabla \varphi \right|$and $-\nabla \cdot \vec{n}=\nabla \cdot \left(\nabla \varphi /\left|\nabla \varphi \right|\right)$. Following Beckermann et al., 1999, we employ a kernel function normal to the interface

$$\begin{array}{c} \varphi =\frac{1-\tanh\left(\frac{\alpha n}{\varepsilon }\right)}{2}, \end{array}$$

wheren is the coordinate normal to the interface so that

$$\begin{array}{l} c=\nabla \cdot \frac{\nabla \varphi }{\left|\nabla \varphi \right|}=\frac{1}{\left|\nabla \varphi \right|}\left(\nabla^{2}\varphi -\frac{\nabla \varphi \cdot \nabla \left|\nabla \varphi \right|}{\left|\nabla \varphi \right|}\right)\\ \;=\frac{1}{\left|\nabla \varphi \right|}\left[\nabla^{2}\varphi -4\frac{\alpha^{2}}{\varepsilon^{2}}\varphi \left(1-\varphi \right)\left(1-2\varphi \right)\right] \end{array}$$

Thus, we arrive at,

$$\begin{array}{c} \tau \frac{\partial \varphi }{\partial t}=\gamma \left(\nabla^{2}\varphi -\frac{G^{\prime }(\varphi )}{\varepsilon^{2}}\right)-\vec{F}\cdot \nabla \varphi , \end{array}$$

where$G=2\alpha^{2}\varphi^{2}{\left(1-\varphi \right)}^{2}$ and $\vec{F}=M_{V}\left(\int \varphi d\vec{r}-V_{0}\right)\nabla \varphi /\left|\nabla \varphi \right|-F_{poly}\nabla \varphi /\left|\nabla \varphi \right|$. The first force term represents the constraints for fixed cell volume and the second term is the force normal to the interface exerted by actin polymerization. Hence, we obtain

$$\begin{array}{l} \tau \frac{\partial \varphi }{\partial t}=\gamma \left(\nabla^{2}\varphi -\frac{G^{\prime }\left(\varphi \right)}{\varepsilon^{2}}\right)-M_{V}\left(V-V_{0}\right)\left|\nabla \varphi \right|+F_{\text{poly}}\left(\vec{r}\right)\left|\nabla \varphi \right|,\\ \vec{v}=-\left[\frac{\gamma \left(\nabla^{2}\varphi -\frac{G^{\prime }\left(\varphi \right)}{\varepsilon^{2}}\right)}{\left|\nabla \varphi \right|}-M_{V}\left(V-V_{0}\right)+F_{\text{poly}}\left(\vec{r}\right)\right]\frac{\nabla \varphi }{\left|\nabla \varphi \right|}. \end{array}$$

#### Kinetic equations for the active patch dynamics

Equations (2) and (3) are derived as follows. For reaction B→A, we assume an autocatalytic reaction following a sigmoidal function with Hill coefficient 2 that is repressed by inhibitor I. For A→B, we assume a constant rate. The inhibitor is produced at the rate $k_{1}A^{2}$ and degraded at a constant rate $k_{2}$. Thus we write,

(Equation S1) $$\begin{array}{l} \frac{\mathrm{d}A}{\mathrm{d}t}=k_{A}\frac{A^{2}B}{K_{A}^{2}+A^{2}}\frac{1}{K_{I}+I}-d_{A}A+D_{A}\nabla^{2}A\\ \frac{\mathrm{d}B}{\mathrm{d}t}=-k_{A}\frac{A^{2}B}{K_{A}^{2}+A^{2}}\frac{1}{K_{I}+I}+d_{A}A+D_{B}\nabla^{2}B\\ \frac{\mathrm{d}I}{\mathrm{d}t}=k_{1}A^{2}-k_{2}I+D_{I}\nabla^{2}I, \end{array}$$

which satisfies the conservation relation

$$\begin{array}{c} \left\langle A\right\rangle +\left\langle B\right\rangle =a_{\text{t}}, \end{array}$$

where⟨⟩ indicates spatial average. By converting the variables to $\tilde{A}=\alpha A/K_{A}$, $\tilde{B}=\alpha B/K_{A}$, $\tilde{I}=I/K_{I}$, $\tilde{t}=d_{A}t$ using a dimensionless parameter $\alpha =\sqrt{k_{A}/K_{I}d_{A}}$, we arrive at

$$\begin{array}{l} \frac{\mathrm{d}\tilde{A}}{\mathrm{d}\tilde{t}}=\frac{{\tilde{A}}^{2}\tilde{B}}{1+{\tilde{A}}^{2}/\alpha^{2}}\frac{1}{1+\tilde{I}}-\tilde{A}+{\tilde{D}}_{A}\nabla^{2}\tilde{A}\\ \frac{\mathrm{d}\tilde{B}}{\mathrm{d}\tilde{t}}=-\frac{{\tilde{A}}^{2}\tilde{B}}{1+{\tilde{A}}^{2}/\alpha^{2}}\frac{1}{1+\tilde{I}}+\tilde{A}+{\tilde{D}}_{B}\nabla^{2}\tilde{B},\\ \frac{\mathrm{d}\tilde{I}}{\mathrm{d}\tilde{t}}={\tilde{k}}_{1}{\tilde{A}}^{2}-{\tilde{k}}_{2}\tilde{I}+{\tilde{D}}_{I}\nabla^{2}\tilde{I}, \end{array}$$

where ${\tilde{D}}_{A}=D_{A}/d_{A}$, ${\tilde{D}}_{B}=D_{B}/d_{A}$, ${\tilde{D}}_{I}=D_{I}/d_{A}$, ${\tilde{k}}_{1}=k_{1}K_{A}^{2}/k_{A}$, ${\tilde{k}}_{2}=k_{2}/d_{A}$. Note that the spatial variable $\vec{r}\left[\text{μm}\right]$ and the diffusion coefficients $\left[\text{μ}{\text{m}}^{2}\right]$ still has dimension. Furthermore we reduce the number of kinetic variables to two by assuming that diffusion of $\tilde{B}$ is sufficiently fast in the timescale of our interest so that $\tilde{B}$ can be approximated as spatially homogeneous. Thus $\tilde{B}$ now becomes enslaved to $\tilde{A}$ according to the conservation relation

$$\begin{array}{c} \left\langle \tilde{A}\right\rangle +\tilde{B}={\tilde{a}}_{\text{t}}, \end{array}$$

where ${\tilde{a}}_{\text{t}}=a_{\text{t}}\alpha /K_{A}$. Equations (2) and (3) are obtained after redefining the variables and the parameters. Note that changes in the parameter in this reduced equation can interpreted by more than one of the original parameters. For example, when the reaction rate in A→B is lowered (i.e., decrease in $d_{A}$), due to mutation in Ras-GAP, $\alpha =\sqrt{k_{A}/K_{I}d_{A}}$ and ${\tilde{a}}_{\text{t}}=a_{\text{t}}\alpha /K_{A}$ increase, which results in enhanced active membrane patch and thus enhanced macropinocytosis and actin wave.

#### Condition for the patch annihilation

In the presence of the inhibitor, the activated spot with high A can annihilates even without coupling with the membrane deformation. Whether the spot in Equations (2) and (3) in the main text annihilates or not depends on the existence of a non-zero stable fixed point in the following equation:

$$\begin{array}{l} \dot{a}=\frac{a^{2}b}{1+a^{2}/\alpha^{2}}\frac{1}{1+I}-a\\ \dot{I}=k_{1}a^{2}-k_{2}I. \end{array}$$

At the fixed point, a satisfies

$$0=\frac{a^{2}b}{1+a^{2}/\alpha^{2}}\frac{1}{1+\kappa a^{2}}-a,$$

where $\kappa =k_{1}/k_{2}$. If and only if the function $f\left(a\right)=ab-\left(1+a^{2}/\alpha^{2}\right)\left(1+\kappa a^{2}\right)$ has a solution f(a)=0 for a>0 and $0\leq b\leq a_{\text{t}}$, the non-zero fixed point exist in Equations (2) and (3). From f(0)<0 and monotonicity of $f^{\prime }\left(a\right)$, the condition for the absence of the non-zero fixed point for $0\leq b\leq a_{\text{t}}$ is given by $f\left(a^{*}\right)<0$, where $a^{*}$ satisfies $f^{\prime }\left(a^{*}\right)=0$. Thus, we obtain

(Equation S2) $$\begin{array}{c} -\frac{1}{2}\left(\frac{1}{\alpha^{2}}+\kappa \right){\left(a^{*}\right)}^{2}+\frac{3}{4}a^{*}b-1<0. \end{array}$$

The sufficient condition for patch annihilation is

$$\begin{array}{c} \kappa >\frac{9}{32}a_{t}^{2}-\frac{1}{\alpha^{2}}. \end{array}$$

Note that neither a non-vanish static spot nor excitable behavior appears if this condition is met. The necessary and sufficient condition for patch annihilation would be obtained by numerically solving $f^{\prime }\left(a^{*}\right)=0.$

#### Numerical simulations

Time evolution of equation for φ, A and I was numerically solved using the standard explicit Euler method with mesh size ⅆx=0.1μm and $\mathrm{d}t=4.0\times 10^{-4}\;\text{ s}$. For A and I, instead of solving Equations (4) and (5), we computed the following equations:

$$\begin{array}{l} \frac{\partial A}{\partial t}=-\nabla \cdot \left(A\vec{v}\right)+D_{A}\beta \left(1-\psi \right)\left(1-2\varphi \right)\nabla \varphi \nabla A+D_{A}\nabla^{2}A+\frac{A^{2}B}{1+A^{2}/\alpha^{2}}\frac{1}{1+I}-A\\ \frac{\partial I}{\partial t}=-\nabla \cdot \left(I\vec{v}\right)+D_{I}\beta \left(1-\psi \right)\left(1-2\varphi \right)\nabla \varphi \nabla I+D_{I}\nabla^{2}I+k_{1}A^{2}-k_{2}I, \end{array}$$

which is derived from the relation $\psi ={\left(1+e^{-\beta \left(\varphi \left(1-\varphi \right)-\theta \right)}\right)}^{-1}$. The above equations were solved for all lattice sites above the cut-off threshold $\psi >10^{-3}$, otherwise A and I were allowed to decay at a rate $\gamma_{2}=10.0$[s^−1^]. Similarly, the equation for $\vec{v}$ in Equation (6) is computed for all sites if $\left|\nabla \varphi \right|>10^{-3}$, otherwise $\vec{v}=0$. In the phase-field framework, the topological change that accompanies membrane fusion occurs naturally by simply solving the partial differential equation Equation (1) without the need for additional numerical implementation. Note that immediately after the cup closure, the internalized cup shrinks and eventually vanishes owing to the surface tension, which causes numerical instability due to an abrupt increase in A on the shrinking membrane. To avoid this instability, an upper limit was set to 50.0, for both A and I. All simulations were coded in C++ with Open-ACC and performed with GPU (NVIDIA GeForce GTX 1080 Ti). Results of three-dimensional simulations were visualized using OpenGL.

#### Volume evaluation of the enclosed extracellular space

Cup closure was judged by evaluating whether the region with φ=0 was surrounded by φ=1. For easier detection of the cup closure and computation involving exhaustive parameter search, we considered a cell shape with z-axis symmetry to run the simulations in a quasi 3-dimensional space with the axisymmetric coordinate (i.e., on a *z*-*r* plane). In this coordinate, $\nabla^{2}$ and $\nabla \cdot \vec{v}$ were replaced by $\nabla^{2}=r^{-1}\frac{\partial }{\partial r}\left(r\frac{\partial }{\partial r}\right)+\frac{\partial }{\partial z}$ and $\nabla \cdot \vec{v}=\frac{1}{r}\frac{\partial }{\partial r}\left(rv_{r}\right)+\frac{\partial }{\partial z}v_{z}$. The Neumann boundary condition $\partial_{r}\varphi =\partial_{r}A=\partial_{r}I=0$ was applied at the boundary r=0, whereas the Dirichlet boundary condition φ=A=I=0 was applied for boundaries at $z=0,\;L_{z}$ and $r=L_{r}$, where $L_{z},\;L_{r}$ are the axial lengths of the system. The analysis consists of two parts (Figure S1G and S1H): (1) scoring of the membrane enclosing events (i.e., whether or not the region with φ=0 that is enclosed by φ=1 exists), and (2) estimating the enclosed volume at the time of cup closure. For the first part, for each simulation time step, the number of transitions from φ=0 to φ=1 (red circles in Figure S1G) was counted along the line r=Δr from (Δr, Lz) to (Δr, 0). By definition, an enclosed region is present when this number is two (Figure S1G, right panel); otherwise, no closure (Figure S1G, left panel). The enclosed volume was estimated at the time of closure by integrating the cross-sectional disk (Figure S1H, left panel), or a disk with a hole at the center (Figure S1H, right panel) at a constant z within $z_{b}\leq z\leq z_{t}$, where $z_{t}$ and $z_{b}$ are the first and second points at which φ changes from φ=0 to 1 (Figure S1H).

#### Simulation with a bead

Cells can engulf a large particle by developing macropinocytic cups in our simulation. To describe a spherical particle (a bead) with radius $R_{b}$ at position $r_{b}$, we introduce a field variable χ(r) that does not change in time as:

$$\chi \left(r\right)=\frac{1+\text{tanh}\left(\frac{R_{b}-\left|r-r_{b}\right|}{\varepsilon /2}\right)}{2}$$

For interaction between membrane and a bead, we numerically solve Equations (1) and (6) with additional terms:

$$\begin{array}{l} \tau \frac{\partial \varphi }{\partial t}=\eta \left(\nabla^{2}\varphi -\frac{G^{\prime }\left(\varphi \right)}{\varepsilon^{2}}\right)-M_{V}\left(V-V_{0}\right)\left|\nabla \varphi \right|+F_{\text{poly}}\left|\nabla \varphi \right|-A_{\text{rep}}\chi^{2}\varphi \\ \;+A_{\text{att}}\left|\nabla \chi \right|\left|\nabla \varphi \right|-\tilde{v}\cdot \nabla \varphi ,\\ v=-\left[\frac{\eta \left(\nabla^{2}\varphi -\frac{G^{\prime }\left(\varphi \right)}{\varepsilon^{2}}\right)}{\left|\nabla \varphi \right|}-M_{V}\left(V-V_{0}\right)+F_{\text{poly}}-A_{\text{rep}}\chi^{2}\varphi +A_{\text{att}}\left|\nabla \chi \right|\left|\nabla \varphi \right|\right]\frac{\nabla \varphi }{\left|\nabla \varphi \right|}+\tilde{v} \end{array}$$

where

$$\tilde{v}=\frac{-1}{\int \varphi \mathrm{d}r}\int \left(r-r_{c}\right)\left[\eta \left(\nabla^{2}\varphi -\frac{G^{\prime }\left(\varphi \right)}{\varepsilon^{2}}\right)-M_{V}\left(V-V_{0}\right)\left|\nabla \varphi \right|+F_{\text{poly}}\left|\nabla \varphi \right|-A_{\text{rep}}\chi^{2}\varphi +A_{\text{att}}\left|\nabla \chi \right|\left|\nabla \varphi \right|\right]\mathrm{d}r.$$

The third and second last terms on the right side of the first equation represent exclusive volume and adhesion effect. The last term is introduced to satisfy the zero net force condition where centroid does not change in time.

For the computation of the phase diagram in Figures5B and 5E, a bead internalization was judged based on quasi 3-dimensional simulations.

#### Reduced model

The proposed model ((Equations 1, 4, 5, 6, and 7) in the main text) assumes that force generated by actin-polymerization occurs at the boundary of the active signaling patch. It is thus expected that as long as there is an expanding circular domain that serves this purpose, the reaction-diffusion process of *A* can be substituted by a simpler ordinary differential equation (ODE) of a growing patch boundary $r_{b}$ (Figure S8A). In the reduced model, the patch expansion along tangential direction of the membrane occurs until the patch size reaches $A_{0}$ which serves as a proxy for $a_{t}$ in the original model. The patch boundary can induce membrane deformation as discussed below. The site of an active protrusion $r_{p}$ is located at distance ${\text{Δ}}_{l}$ along the membrane from $r_{b}$ (see Figure S8A).

The patch boundary $r_{b}$ moves together with the membrane and thus its velocity in the direction normal to the membrane is the same as that of the membrane deformation v (Equation (6) main text). $r_{b}$also moves in the tangential direction to the membrane, which represents expansion or shrinkage of the active patch. Here, we adopt the following equation of $r_{b}$ in the z axis-symmetric coordinate (i.e., on a *z*-*r* plane).

$$\frac{\partial }{\partial t}r_{b}=v+v_{t}T\left(A-A_{0}\right)$$

The second term in the right hand side represents expansion or shrinkage of the active patch: ***T*** denotes unit tangential vector to the membrane $T=\left(\partial_{z}\varphi /\left|\nabla \varphi \right|,-\partial_{r}\varphi /\left|\nabla \varphi \right|\right)$ and $v_{t}$ is a positive constant that determines the tangential velocity. *A* is a normalized area of the active patch encircled by $r_{b}$ (FigureS8A) and is numerically computed by the following path integral along T:

$$A=\frac{2\pi \int_{C}rds}{4\pi R_{0}^{2}}$$

where *C* is path along T from $r_{b}$ to the point of r=0. The equation of $r_{b}$ and Equation (1) in the main text are numerically solved by applying

$$F_{\text{poly}}\left(r,r_{p}\right)=f\left(r_{f}-\left|r-r_{p}\right|\right),$$

where $r_{f}$ is width of the protrusive site. $\;r_{p}$was obtained numerically by the integral of−T so that the length along the membrane from $r_{b}$ to $r_{p}$ is ${\text{Δ}}_{l}$. f(x)is a sigmoidal function $f\left(x\right)={\left[1+\exp\left(-2.5x\right)\right]}^{-1}$.

The essential behaviors of cup formation and closure are reproduced in the reduced model (Figures S10B–S10E). Qualitative features still holds; for a large F/η the cups successfully close and enclose a large extracellular volume (Figure S8C), whereas for small F/η the cup fails to close (Figure S8D). At intermediate value of F/η, cups form repetitively (Figure S8E) as was observed in the original model. This demonstrates that the essential ingredient of micropinocytosis is the dynamics of the patch boundary rather than reaction-diffusion process itself. The phase diagram of the reduced model (Figure S8B) does not align exactly with that of the original model due to simplification such as linear dependency of the patch velocity on *A* and an assumption of infinitely sharp interface of the patch boundary.

**Table:** Table for model parameters

Parameters	Description	Equation	Value
$A_{t}$	The total number of ‘A’ and ‘B’ molecules	–	-[the unit of number of molecules]
$a_{t}$	$A_{t}\;$normalized by the minimum surface area $a_{\text{t}}=A_{\text{t}}/4\pi R_{0}^{2}$.	–	1.94, 2.40–2.90 [*c*: the unit of concentration]
α	The half-maximal concentration for the autocatalytic reaction B→A.	Equation (2)	1–∞ [*c*]
$k_{1}$	The production rate of ‘I’.	Equation (3)	0, 2.0 × 10^−4^, 0.088 [s^−1^ c^−2^]
$k_{2}$	The degradation rate of ‘I’.	Equation (3)	2.0 × 10^−4^, 0.54 [s^−1^ c^−1^]
$D_{A}$	The diffusion constant of ‘A’.	Equation (2)	0.085, 0.10, 0.26 [μm^2^/s]
$D_{I}$	The diffusion constant of ‘I’.	Equation (3)	0.01, 0.11, 0.87 [μm^2^/s]
η	The cell surface tension.	Equation (1)	0.5 [n*N*/μm]; Ref. (Álvarez-González et al., 2015)
ε	The effective thickness of the simulated membrane.	Equation (1)	0.8 [μm]
$M_{V}$	The volume constraint parameter.	Equation (1)	5.0 [n*N*/μm^5^]
$R_{0}$	The cell radius	–	10.0 [μm]
$V_{0}$	The cell volume.	Equation (1)	$4\pi \times 10.0^{3}/3\;\left[\text{μ}{\text{m}}^{3}\right]$
τ	The deformation timescale (inverse of the membrane velocity per unit stress).	Equation (1)	5, 7, 10, 20 [sec. n*N*/μm^3^]
F	The force magnitude scaled by the spatial coarsening factor 1/3 (see text).	Equation (7)	0.8–2.8 [nN/μm^2^]; Ref (Abraham et al., 1999)
K and $K^{\prime }$	The half-maximal concentration of ‘A’ that induces (K) or suppresses ($K^{\prime }$) force generation.	Equations (7) and (9)	0.005, 0.01, 0.086 [*c*](*K*), and 0.1,0.25, 1.8[*c*] (*K*′)
$n_{h}$	The Hill coefficient in the force generation.	Equations (7) and (9)	3, 5
$K_{b}$	Bending modulus.	–	1.6 pNμm; Ref.(Simson et al., 1998)
β and θ	The sharpness and the width of the interface ψ.	–	100.0 (β) 0.105 (θ)
$r_{init}$	The initial seed for the patch radius.	–	1.0, 1.5 [μm]

### Quantification and statistical analysis

No statistical analysis is used.

## Data Availability

• All data reported in this paper will be shared by the lead contact upon request. • Original codes has been deposited at [https://github.com/nen6f/3D-simulations-of-macropinocytosis] and is publicly available as of the date of publication. DOIs for the software for the visualization and the parameter values in “Table for model parameters” are listed in the key resources table. • Any additional information required to reanalyze the data reported in this paper is available from the lead contact upon request.

## References

[bib1] Abraham V.C., Krishnamurthi V., Taylor D.L., Lanni F. (1999). The actin-based nanomachine at the leading edge of migrating cells. Biophysical J..

[bib2] Aguado-Velasco C., Bretscher M.S. (1999). Circulation of the plasma membrane in Dictyostelium. Mol. Biol. Cell.

[bib3] Álvarez-González B., Meili R., Bastounis E., Firtel R.A., Lasheras J.C., Del Álamo J.C. (2015). Three-dimensional balance of cortical tension and axial contractility enables fast amoeboid migration. Biophysical J..

[bib4] Antkowiak A., Guillotin A., Boiero Sanders M., Colombo J., Vincentelli R., Michelot A. (2019). Sizes of actin networks sharing a common environment are determined by the relative rates of assembly. PLoS Biol..

[bib5] Araki N., Hatae T., Yamada T., Hirohashi S. (2000). Actinin-4 is preferentially involved in circular ruffling and macropinocytosis in mouse macrophages: analysis by fluorescence ratio imaging. J. Cell Sci..

[bib7] Bar-Sagi D., Feramisco J.R. (1986). Induction of membrane ruffling and fluid-phase pinocytosis in quiescent fibroblasts by ras proteins. Science.

[bib19] Beckermann C., Diepers H.-J., Steinbach I., Karma A., Tong X. (1999). Modeling melt convection in phase-field simulations of solidificatio. J. Comput. Phys..

[bib8] Bernitt E., Döbereiner H.G., Gov N.S., Yochelis A. (2017). Fronts and waves of actin polymerization in a bistability-based mechanism of circular dorsal ruffles. Nat. Commun..

[bib9] Bleicher P., Sciortino A., Bausch A.R. (2020). The dynamics of actin network turnover is self-organized by a growth-depletion feedback. Sci. Rep..

[bib11] Bloomfield G., Kay R.R. (2016). Uses and abuses of macropinocytosis. J. Cell Sci..

[bib10] Bloomfield G., Traynor D., Sander S.P., Veltman D.M., Pachebat J.A., Kay R.R. (2015). Neurofibromin controls macropinocytosis and phagocytosis in Dictyostelium. ELife.

[bib12] BoseDasgupta S., Pieters J. (2014). Inflammatory stimuli reprogram macrophage phagocytosis to macropinocytosis for the rapid elimination of pathogens. *PLoS Pathog.*.

[bib14] Bretschneider T., Anderson K., Ecke M., Müller-Taubenberger A., Schroth-Diez B., Ishikawa-Ankerhold H.C., Gerisch G. (2009). The three-dimensional dynamics of actin waves, a model of cytoskeletal self-organization. Biophysical J..

[bib13] Bretschneider T., Diez S., Anderson K., Heuser J., Clarke M., Müller-Taubenberger A., Köhler J., Gerisch G. (2004). Dynamic actin patterns and Arp2/3 assembly at the substrate-attached surface of motile cells. Curr. Biol..

[bib15] Brzeska H., Koech H., Pridham K.J., Korn E.D., Titus M.A. (2016). Selective localization of myosin-I proteins in macropinosomes and actin waves. Cytoskeleton.

[bib17] Buckley C.M., King J.S. (2017). Drinking problems: mechanisms of macropinosome formation and maturation. FEBS J..

[bib16] Buckley C.M., Pots H., Gueho A., Vines J.H., Munn C.J., Phillips B.A., Gilsbach B., Traynor D., Nikolaev A., Soldati T. (2020). Coordinated Ras and Rac activity shapes macropinocytic cups and enables phagocytosis of geometrically diverse bacteria’. Curr. Biol..

[bib18] Burke T.A., Christensen J.R., Barone E., Suarez C., Sirotkin V., Kovar D.R. (2014). Homeostatic actin cytoskeleton networks are regulated by assembly factor competition for monomers. Curr. Biol..

[bib20] Camley B.A., Zhao Y., Li B., Levine H., Rappel W.J. (2017). Crawling and turning in a minimal reaction-diffusion cell motility model: coupling cell shape and biochemistry. Phys. Rev. E.

[bib21] Campbell E.J., Bagchi P. (2018). A computational model of amoeboid cell motility in the presence of obstacles. Soft Matter.

[bib22] Cao Y., Ghabache E., Miao Y., Niman C., Hakozaki H., Reck-Peterson S.L., Devreotes P.N., Rappel W.J. (2019). A minimal computational model for three-dimensional cell migration. J. R. Soc. Interf..

[bib23] Carlier M.F., Shekhar S. (2017). Global treadmilling coordinates actin turnover and controls the size of actin networks. Nat. Rev. Mol. Cell Biol..

[bib24] Chabaud M., Heuze M.L., Bretou M., Vargas P., Maiuri P., Solanes P., Maurin M., Terriac E., Le Berre M., Lankar D. (2015). Cell migration and antigen capture are antagonistic processes coupled by myosin II in dendritic cells. Nat. Commun..

[bib25] Chubb J.R., Wilkins A., Thomas G.M., Insall R.H. (2000). The Dictyostelium RasS protein is required for macropinocytosis, phagocytosis and the control of cell movement. J. Cell Sci..

[bib26] Commisso C., Davidson S.M., Soydaner-Azeloglu R.G., Parker S.J., Kamphorst J.J., Hackett S., Grabocka E., Nofal M., Drebin J.A., Thompson C.B. (2013). Macropinocytosis of protein is an amino acid supply route in Ras-transformed cells. Nature.

[bib27] Condon N.D., Heddleston J.M., Chew T.L., Luo L., McPherson P.S., Ioannou M.S., Hodgson L., Stow J.L., Wall A.A. (2018). Macropinosome formation by tent pole ruffling in macrophages. J. Cell Biol..

[bib28] Dai J., Ting-Beall H.P., Hochmuth R.M., Sheetz M.P., Titus M.A. (1999). Myosin I contributes to the generation of resting cortical tension. Biophysical J..

[bib6] De Baey A., Lanzavecchia A. (2000). The role of aquaporins in dendritic cell macropinocytosis. J. Exp. Med..

[bib43] De Hostos E.L. (1999). The coronin family of actin-associated proteins. Trends Cell Biol..

[bib29] Diegmiller R., Montanelli H., Muratov C.B., Shvartsman S.Y. (2018). Spherical caps in cell polarization. Biophysical J..

[bib30] Flemming S., Font F., Alonso S., Beta C. (2020). How cortical waves drive fission of motile cells. Proc. Natl. Acad. Sci. U S A.

[bib31] Frost A., Perera R., Roux A., Spasov K., Destaing O., Egelman E.H., De Camilli P., Unger V.M. (2008). Structural basis of membrane invagination by F-BAR domains. Cell.

[bib32] Funamoto S., Meili R., Lee S., Parry L., Firtel R.A. (2002). Spatial and temporal regulation of 3-phosphoinositides by PI 3-kinase and PTEN mediates chemotaxis. Cell.

[bib33] Gerhardt M., Ecke M., Walz M., Stengl A., Beta C., Gerisch G. (2014). Actin and PIP3 waves in giant cells reveal the inherent length scale of an excited state. J. Cell Sci..

[bib34] Gerisch G., Schroth-Diez B., Müller-Taubenberger A., Ecke M. (2012). PIP3 waves and PTEN dynamics in the emergence of cell polarity. Biophysical J..

[bib35] Goode B.L., Wong J.J., Butty A.C., Peter M., McCormack A.L., Yates J.R., Drubin D.G., Barnes G. (1999). Coronin promotes the rapid assembly and cross-linking of actin filaments and may link the actin and microtubule cytoskeletons in yeast. J. Cell Biol..

[bib36] Hacker U., Albrecht R., Maniak M. (1997). Fluid-phase uptake by macropinocytosis in dictyostelium. J. Cell Sci..

[bib37] Hecht I., Kessler D.A., Levine H. (2010). Transient localized patterns in noise-driven reaction-diffusion systems. Phys. Rev. Lett..

[bib38] Helenius J., Ecke M., Müller D.J., Gerisch G. (2018). Oscillatory switches of Dorso-ventral polarity in cells confined between two surfaces. Biophysical J..

[bib39] Herant M., Heinrich V., Dembo M. (2006). Mechanics of neurophil phagocytosis: experiments and quantitative models. J. Cell Sci..

[bib40] Hewlett L.J., Prescott A.R., Watts C. (1994). The coated pit and macropinocytic pathways serve distinct endosome populations. J. Cell Biol..

[bib41] Hoeller O., Bolourani P., Clark J., Stephens L.R., Hawkins P.T., Weiner O.D., Weeks G., Kay R.R. (2013). Two distinct functions for PI3-kinases in macropinocytosis. J. Cell Sci..

[bib42] Honda G., Saito N., Fujimori T., Hashimura H., Nakamura M.J., Nakajima A., Sawai S. (2020). Micro-topographical guidance of macropinocytic signaling patches. bioRxiv.

[bib44] Inaba H., Yoda K., Adachi H. (2017). The F-actin-binding RapGEF GflB is required for efficient macropinocytosis in Dictyostelium. J. Cell Sci..

[bib45] Itoh T., Hasegawa J. (2013). Mechanistic insights into the regulation of circular dorsal ruffle formation. J. Biochem..

[bib46] Jasnin M., Beck F., Ecke M., Fukuda Y., Martinez-Sanchez A., Baumeister W., Gerisch G. (2019). The architecture of traveling actin waves revealed by cryo-electron tomography. Structure.

[bib47] Kabayama H., Takeuchi M., Taniguchi M., Tokushige N., Kozaki S., Mizutani A., Nakamura T., Mikoshiba K. (2011). Syntaxin 1B suppresses macropinocytosis and semaphorin 3A-induced growth cone collapse. J. Neurosci..

[bib48] Kaksonen M., Roux A. (2018). Mechanisms of clathrin-mediated endocytosis. Nat. Rev. Mol. Cell Biol..

[bib49] Kamphorst J.J., Nofal M., Commisso C., Hackett S.R., Lu W., Grabocka E., Vander Heiden M.G., Miller G., Drebin J.A., Bar-Sagi D. (2015). Human pancreatic cancer tumors are nutrient poor and tumor cells actively scavenge extracellular protein. Cancer Res..

[bib50] Karma A., Rappel W.-J. (1998). Quantitative phase-field modeling of dendritic growth in two and three dimensions. Phys. Rev. E.

[bib51] King J.S., Kay R.R. (2019). The origins and evolution of macropinocytosis. Phil. Trans. R. Soc. B.

[bib52] Kockelkoren J., Levine H., Rappel W.-J. (2003). Computational approach for modeling intra- and extracellular dynamics. Phys. Rev. E.

[bib53] Lee J. (2018). Insights into cell motility provided by the iterative use of mathematical modeling and experimentation. AIMS Biophys..

[bib54] Levine H., Rappel W.J. (2005). Membrane-bound turing patterns. Phys. Rev. E.

[bib55] Lomakin A.J., Lee K.C., Han S.J., Bui D.A., Davidson M., Mogilner A., Danuser G. (2015). Competition for actin between two distinct F-actin networks defines a bistable switch for cell polarization. Nat. Cell Biol..

[bib56] Lowengrub J., Allard J., Aland S. (2016). Numerical simulation of endocytosis: viscous flow driven by membranes with non-uniformly distributed curvature-inducing molecules. J. Comput. Phys..

[bib57] Lowengrub J.S., Rätz A., Voigt A. (2009). Phase-field modeling of the dynamics of multicomponent vesicles: spinodal decomposition, coarsening, budding, and fission. Phys. Rev. E.

[bib58] Meinhardt H. (1999). Orientation of chemotactic cells and growth cones: models and mechanisms. J. Cell Sci..

[bib59] Mercer J., Helenius A. (2009). Virus entry by macropinocytosis. Nat. Cell Biol..

[bib60] Miao Y., Bhattacharya S., Banerjee T., Abubaker-sharif B., Long Y., Inoue T., Iglesias P.A., Devreotes P.N. (2019). Wave patterns organize cellular protrusions and control cortical dynamics.. Mol. Syst. Biol..

[bib61] Mori Y., Jilkine A., Edelstein-Keshet L. (2008). Wave-pinning and cell polarity from a bistable reaction-diffusion system. Biophysical J..

[bib62] Moure A., Gomez H. (2016). Computational model for amoeboid motion: coupling membrane and cytosol dynamics. Phys. Rev. E.

[bib63] Najem S., Grant M. (2013). A phase field model for neural cell chemotropism. Europhys. Lett..

[bib64] Noguchi H. (2016). Membrane tubule formation by banana-shaped proteins with or without transient network structure. Sci. Rep..

[bib65] Norbury C.C. (2006). Drinking a lot is good for dendritic cells. Immunology.

[bib66] Richards D.M., Endres R.G. (2017). How cells engulf: a review of theoretical approaches to phagocytosis. Rep. Prog. Phys..

[bib67] Rueda-Contreras M.D., Romero-Arias J.R., Aragon J.L., Barrio R.A. (2018). Curvature-driven spatial patterns in growing 3D domains: a mechanochemical model for phyllotaxis. PLoS ONE.

[bib68] Sadeghi M., Noé F. (2020). Large-scale simulation of biomembranes incorporating realistic kinetics into coarse-grained models. Nat. Commun..

[bib69] Sasaki A.T., Janetopoulos C., Lee S., Charest P.G., Takeda K., Sundheimer L.W., Meili R., Devreotes P.N., Firtel R.A. (2007). G protein-independent Ras/PI3K/F-actin circuit regulates basic cell motility. J. Cell Biol..

[bib70] Shao D., Levine H., Rappel W.J. (2012). Coupling actin flow, adhesion, and morphology in a computational cell motility model. Proc. Natl. Acad. Sci. USA.

[bib71] Shao D., Rappel W.J., Levine H. (2010). Computational model for cell morphodynamics. Phys. Rev. Lett..

[bib72] Simson R., Wallraff E., Faix J., Niewöhner J., Gerisch G., Sackmann E. (1998). Membrane bending modulus and adhesion energy of wild-type and mutant cells of Dictyostelium lacking talin or cortexillins. Biophysical J..

[bib73] Suarez C., Kovar D.R. (2016). Internetwork competition for monomers governs actin cytoskeleton organization. Nat. Rev. Mol. Cell Biol..

[bib74] Swanson J.A. (2008). Shaping cups into phagosomes and macropinosomes. Nat. Rev. Mol. Cell Biol..

[bib75] Swanson J.A., Yoshida S. (2019). Macropinosomes as units of signal transduction. Phil. Trans. R. Soc. B.

[bib76] Taniguchi D., Ishihara S., Oonuki T., Honda-Kitahara M., Kaneko K., Sawai S. (2013). Phase geometries of two-dimensional excitable waves govern self-organized morphodynamics of amoeboid cells. Proc. Natl. Acad. Sci. USA.

[bib77] Tjhung E., Tiribocchi A., Marenduzzo D., Cates M.E. (2015). A minimal physical model captures the shapes of crawling cells. Nat. Commun..

[bib78] Traynor D., Kay R.R. (2007). Possible roles of the endocytic cycle in cell motility. J. Cell Sci..

[bib79] Veltman D.M., Lemieux M.G., Knecht D.A., Insall R.H. (2014). PIP3-dependent macropinocytosis is incompatible with chemotaxis. J. Cell Biol..

[bib80] Veltman D.M., Williams T.D., Bloomfield G., Chen B.C., Betzig E., Insall R.H., Kay R.R. (2016). A plasma membrane template for macropinocytic cups. eLife.

[bib81] Weiner O.D., Marganski W.A., Wu L.F., Altschuler S.J., Kirschner M.W. (2007). An actin-based wave generator organizes cell motility. PLoS Biol..

[bib82] Wigbers M.C., Brauns F., Hermann T., Frey E. (2020). Pattern localization to a domain edge. Phys. Rev. E.

[bib83] Williams T.D., Peak-Chew S.Y., Paschke P., Kay R.R. (2019). Akt and SGK protein kinases are required for efficient feeding by macropinocytosis. J. Cel. Sci..

[bib84] Williams T.D., Paschke P.I., Kay R.R. (2019). Function of small GTPases in Dictyostelium macropinocytosis. Phil. Trans. R. Soc. B.

[bib85] Yerbury J.J. (2016). Protein aggregates stimulate macropinocytosis facilitating their propagation. Prion.

[bib86] Yoshida S., Hoppe A.D., Araki N., Swanson J.A. (2009). Sequential signaling in plasma-membrane domains during macropinosome formation in macrophages. J. Cell Sci..

