# Supplementary material for: Three-dimensional morphodynamic simulations of macropinocytic cups
Source: iScience. 2021 Oct 1;24(10):103087. doi: 10.1016/j.isci.2021.103087 (PMC8560551; doi:10.1016/j.isci.2021.103087)
Supplement: Figures S1–S9 [file mmc1.pdf]

iScience, Volume *24*

Supplemental information

Three-dimensional morphodynamic simulations of macropinocytic cups

Nen Saito and Satoshi Sawai

Supplemental information

Supplemental Figure Legends

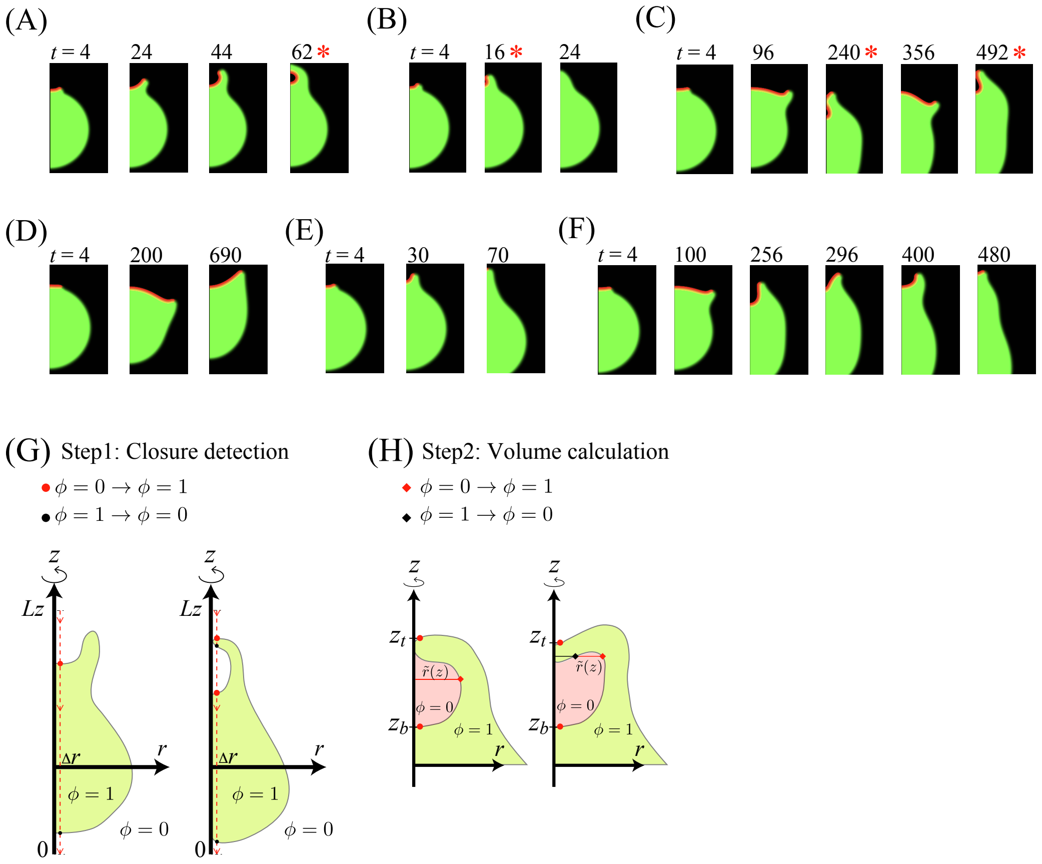

Figure S1. Calculation of the enclosed volume, Related to Figure 2. (*A-F*) Time course of the active patch development in a quasi-3 dimensional space with z-axis symmetry. Parameters correspond to those used in (A) Figure 2*A*, (B) Figure 2*C*, (C) Figure 2*F*, (D) Figure 2*D*, (E) Figure 2*E*, and (F) Figure 2*G*. (*G*, *H*) Calculation of the enclosed volume in the z-axis symmetric coordinate: (G) Detection of cup closure. The number of transitions from $\phi = 0$ to 1 (red circles) was counted along the linear path $r = \Delta r$ from (Δr, Lz) to (Δr, 0) where Δr is the simulation mesh size (red dotted line). The first occurrence of 2 transitions was scored as the time of closure (right panel). (*H*) The enclosed volume was calculated by integrating the cross sectional area within $z_b \leq z \leq z_t$ (red shaded region).

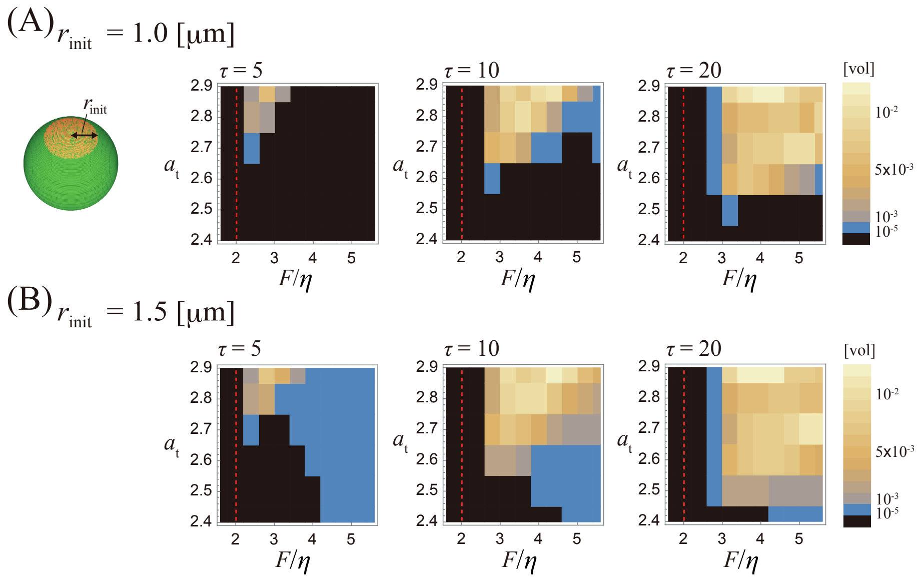

Figure S2. Cup formation and closure depend on the balance between the patch size and the strength of the protrusion force, Related to Figure 2. Phase diagram before averaging over r_{init}. (*A*) $r_{\mathrm{init}} = 1.0$ and (*B*) $r_{\mathrm{init}} = 1.5$. Colors represent the average enclosed volume normalized by the total cell volume (blue to orange). Averages were obtained from three independent simulation runs. Enclosure of volume fraction smaller than 10^{-5} was scored as no uptake (black). Other parameters are the same as in Figure 2.

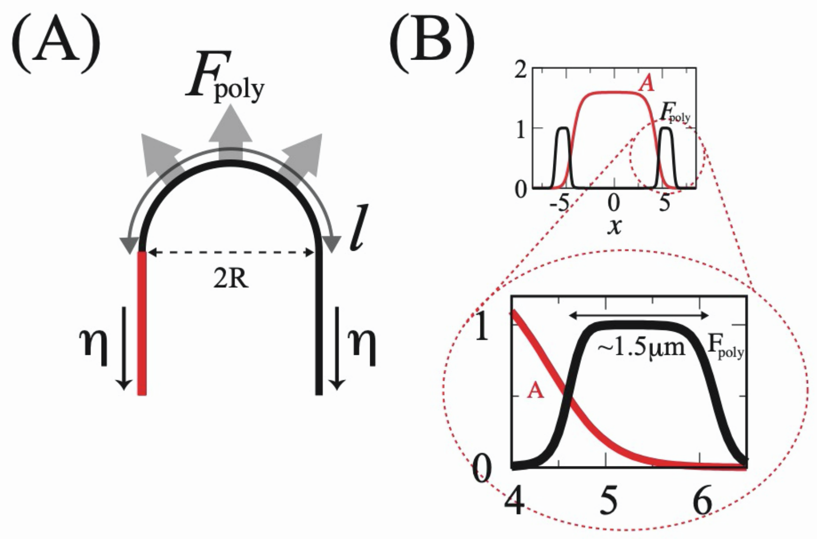

Figure S3. Outward protrusion force required to sustain the cup-shaped membrane protrusion can be estimated from an approximated edge geometry, Related to Figure 2. (*A*) Force profile along the mid-line cross section of an idealized protrusion with width *2R* that consists the rim of the cup. Outward active force per unit length F_{poly} and the line tension η are exerted on the semicircular head of length $l_h = \pi R$. Red represents a region with high A. (*B*) A blow-up view of the force distribution in Figure 1*E*. A snapshot from quasi-2 dimensional space simulation of Eqs. (2) and (3) ($a_t = 2.6$, $k_1 = k_2 = 0$, $\alpha = 1.0$, $D_a = 0.1$ and $D_i = 0.01$) without membrane deformation. Force in a direction normal to the membrane is exerted at the outer edge of width l (a high A region) ($F_{\mathrm{poly}}(A) \simeq F$ in Eq.(7)). $l_h \sim 1.5$ [μm] for $K = 0.005$, $K' = 0.25$ and $n_{\mathrm{h}} = 3$.

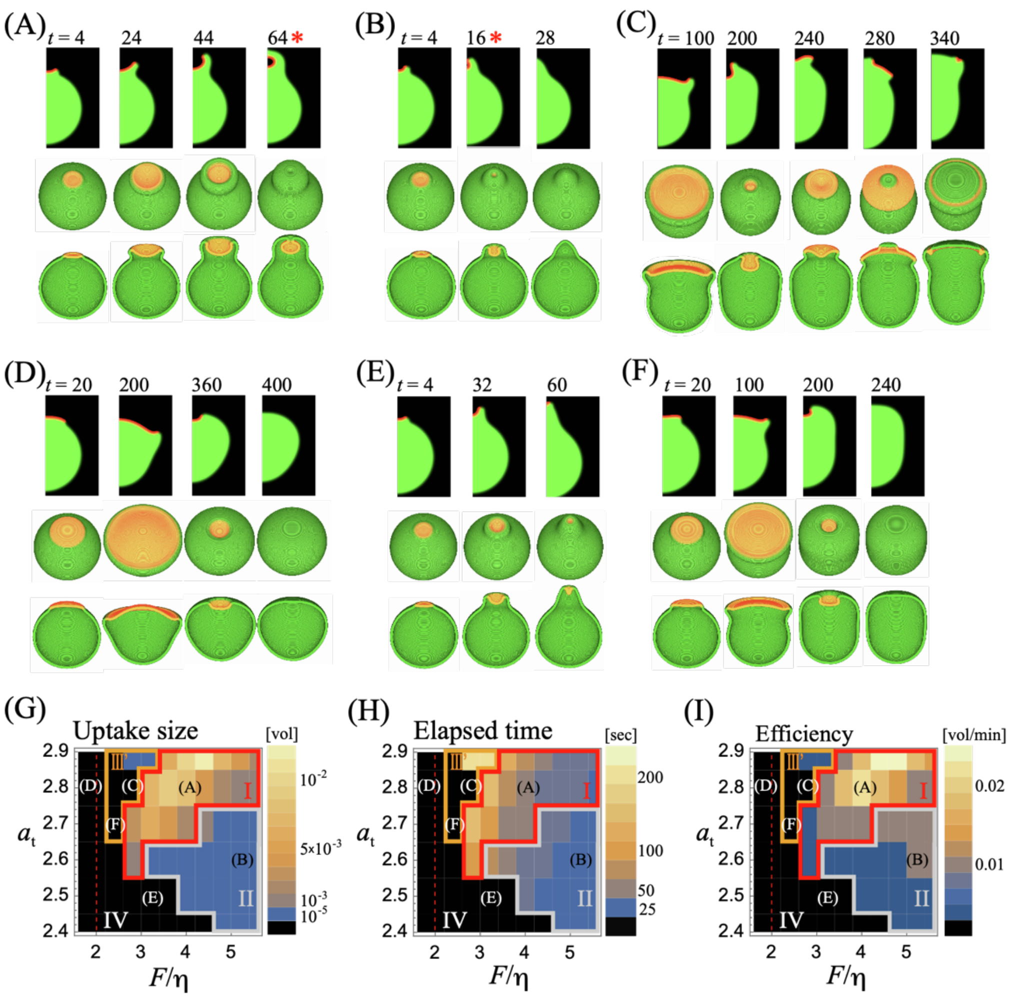

Figure S4. The inhibitor suppresses the repetitive cup formation at Phase III, Related to Figure 2 and 3. ($k_1 = k_2 = 2.0 \times 10^{-4}$) (*A-F*) Representative timeseries for $\tau = 10$. (*A*) $F/\eta = 4.0$, $a_t = 2.8$, (*B*) $F/\eta = 5.2$, $a_t = 2.6$, (*C*) $F/\eta = 2.8$, $a_t = 2.8$, (*D*) $F/\eta = 1.6$, $a_t = 2.8$, (*E*) $F/\eta = 3.2$, $a_t = 2.5$, (*F*) $F/\eta = 2.4$, $a_t = 2.7$. Other parameters are the same as in Figure 2. Simulations in quasi-3 dimensional space (top panels). The top and the cross section view of the full-3 dimensional simulations (middle and bottom panels). Asterisks represents the time of cup closure. (*G-I*) Phase diagram of the cup dynamics. The enclosed volume normalized by the cell size (G), the elapsed time between the patch initiation and cup closure (*H*), and the efficiency (*I*). The averages were taken from six independent simulations (three of each $r_{\text{init}} = 1.0$ μm and 1.5 μm). The

enclosed volume fraction smaller than 10^{-5} was scored as no uptake. For (*H*) and (*I*), the average was taken from the samples showing successful cup closure.

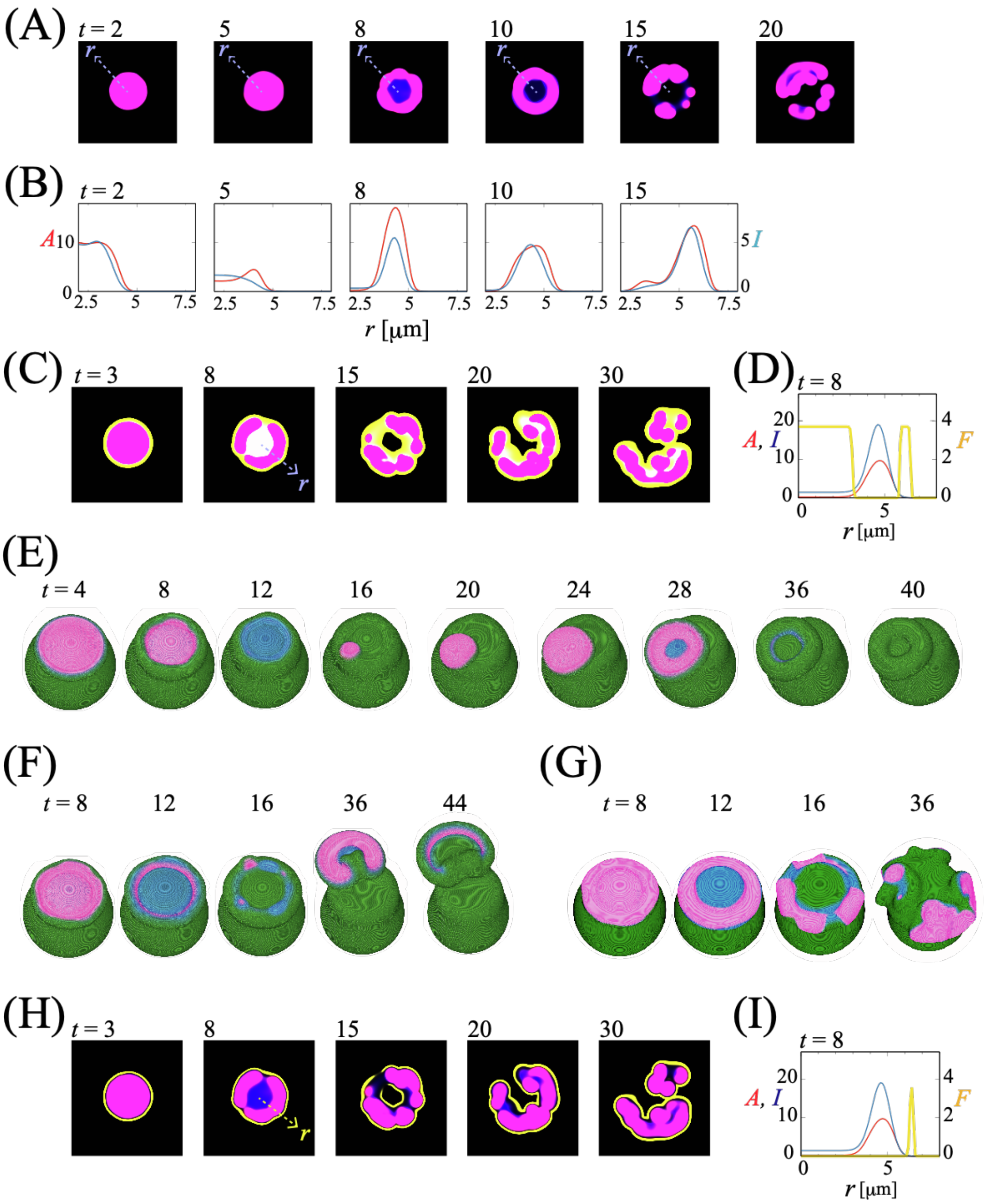

Figure S5. Splitting of patches and cups occurs via a destabilized wavefront in the presence of an inhibitor, Related to Figure 6. (*A-D*) Representative reaction dynamics

on a flat 2-D space in the absence of membrane deformation for $a_t = 1.985$, $k_1 = 0.088$, $k_2 = 0.54, D_a = 0.085$, $D_i = 0.11$. Snapshots (*A*) and the cross-sectional profile (*B*) along the white dashed line in (A). (*C*, *D*) Imaginary force distribution computed according to F_{poly} with A -dependency (Eq. (7)) (*C*; yellow). The spatial profile along the white line (*C*; *t* = 8)(*D*). (*E*-*G*) Representative membrane deformation simulations with Eq. (7); $F = 3.7$ (*E*), $F = 3.0$ (*F*) and $F = 2.0$ (*G*). Other parameters are same to Figure 6*C*. (*H*, *I*) Force distribution calculated according to protrusion force with *A* and I -dependency (Eq. (9)) (*H*; yellow). Snapshots (*H*) and the cross-sectional profile (*I*) along the white line (*H*; *t* = 8). Colors in flat plane simulations indicate regions with overlapping *A* and *I* (pink) and *I* alone (blue), respectively (*A*, *C*, *H*). The color scheme for 3D simulations are the same as in Figure 6.

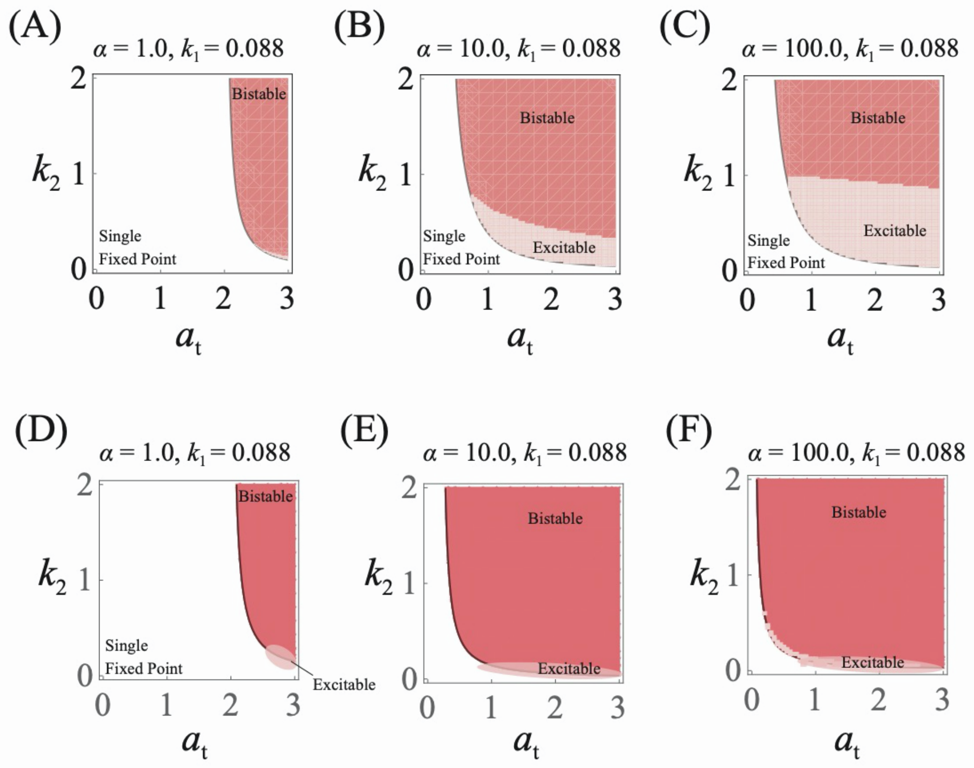

Figure S6. The excitable domain increases in the parameter space for large α, Related to Figure 6. (*A*-*C*) Phase diagrams for Eqs. (2) and (3) at $k_1 = 0.088$ and $\alpha =$ 1.0 (*A*), 10.0 (*B*) and 100.0 (*C*). Mono-stable (white): a patch vanishes and the spatially uniform state is stable at the steady state. Bistable (red): a spatial domain with high A coexist with low A domain at the steady state. Excitable (pink): small perturbation elicits a large increase in A before returning to low A steady state.

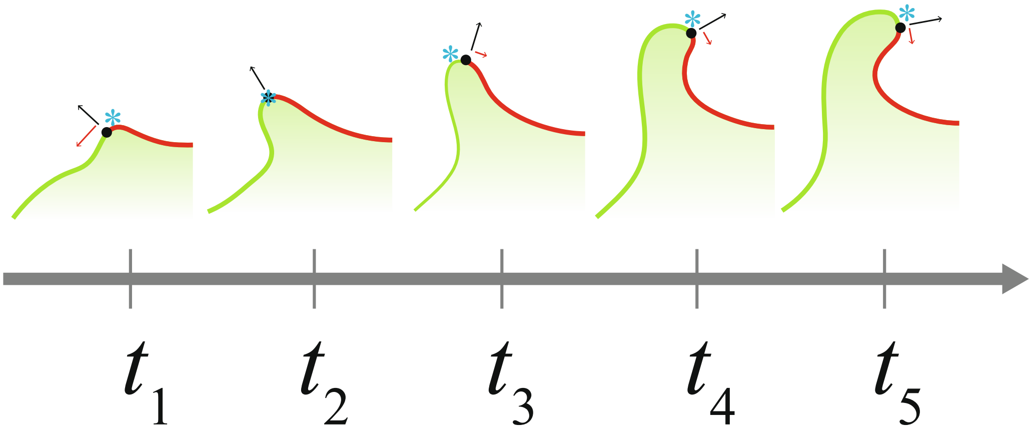

Figure S7. The edge of the active patch is displaced from the cup ridge due to the limiting patch factor, Related to Figure 2. The protruding force is exerted at the edge (black circle) of a self-organized active patch (red). The protrusive force is normal to the membrane (black arrows). Expansion of an active patch ($t = t_1$; red arrow) slows down as the patch size grow and slightly exceeds its maximum size limit ($t = t_2$). As the patch begins to shrink and restores the maximum size, the edge of the patch (black circle) is slightly displaced towards the inner side of the cup, rather than at the very rim (highest curvature region, blue asterisk) ($t = t_3$, t_4, t_5; red arrows). When the protrusion wins over the surface tension that tries to restore high curvature in the inner cup region, the inward extension can grow to enclose a large volume of extracellular fluid until the edges meet and closes the cup.

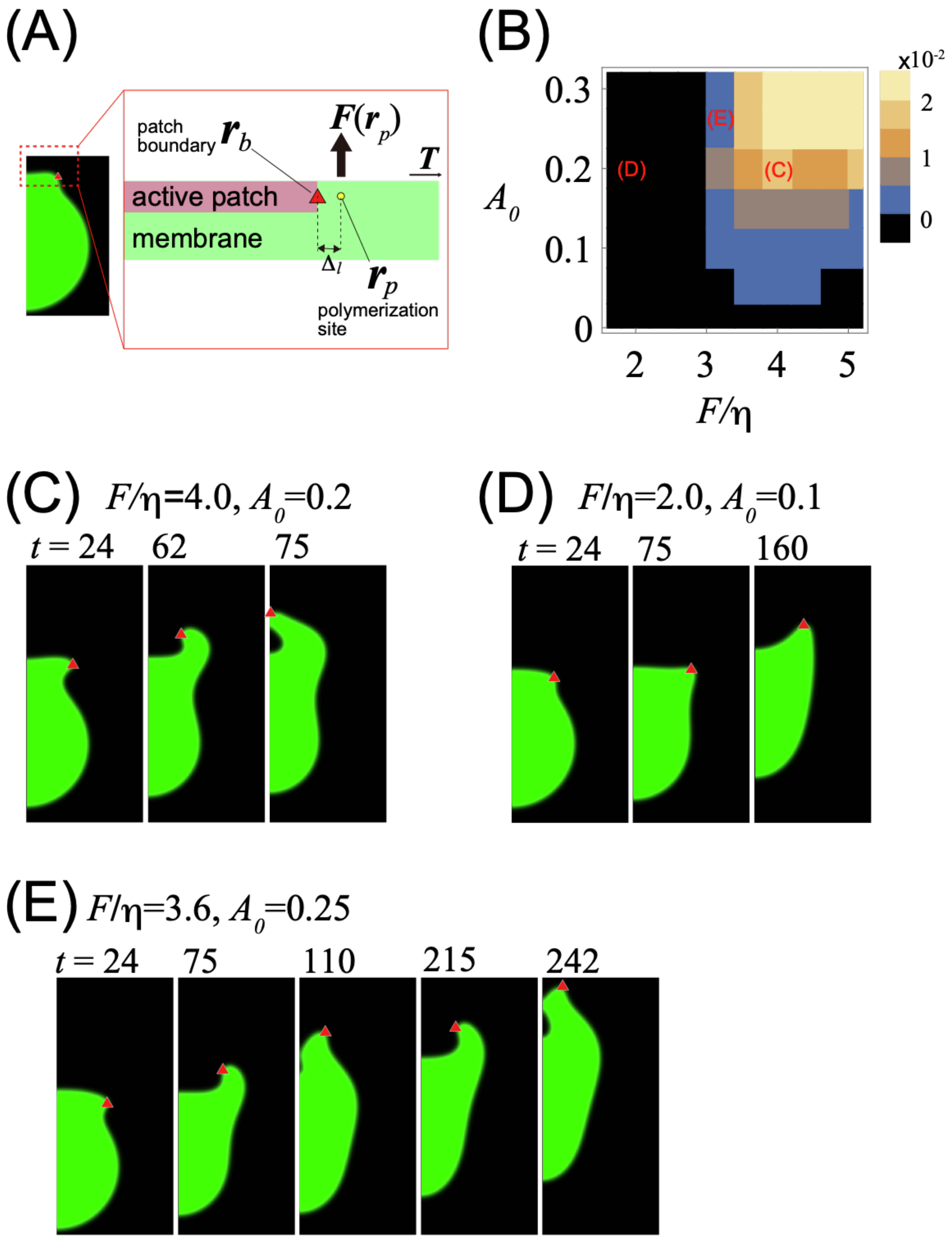

Figure S8. The reduced model captures the essential cup dynamics, Related to Figure 2. (*A*) A schematic of the reduced model. The protrusion site $\boldsymbol{r_p}$ on the membrane

is located outside of the patch Δ_l away from the boundary $\boldsymbol{r_b}$. The protrusive force F_{poly} normal to the membrane (bold arrow). (*B*) Phase diagram. Color bars: the volume of enclosure normalized by the cell size. Parameter sets for (*C*), (*D*) and (*E*) are indicated in the diagram. (*C-E*) Representative time course of membrane deformation in a quasi-3 dimensional space with z-axis symmetry for (*C*) $F/\eta = 4.0$, $A_0 = 0.2$, (*D*) $F/\eta = 2.0$, $A_0 = 0.1$, (*E*) $F/\eta = 3.6$, $A_0 = 0.25$. Red triangles represent the position of $\boldsymbol{r_b}$. Other parameters: $\tau = 2.5$, $\varepsilon = 0.8$, $M_V = 5.0$, $\eta = 0.5$, $v_t = 1.3$, $r_f = 0.75$, $\Delta_l = 0.5$.

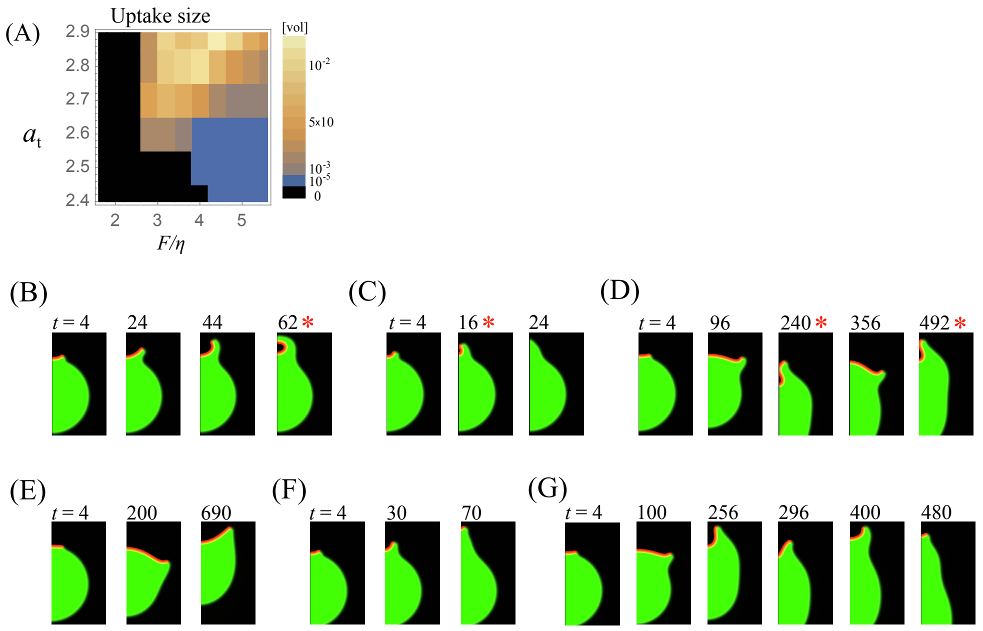

Figure S9. Simulations with the bending energy, Related to Figure 2. Simulation results with the bending modulus term $-K_b\left(\nabla^2 - \frac{G''}{\epsilon^2}\right)\left(\nabla^2\phi - \frac{G'(\phi)}{\epsilon^2}\right)$ (Shao, Rappel and Levine, 2010; Shao, Levine and Rappel, 2012b) included in the r.h.s of Eq.(1). $K_b = 0.0016$ nN · µm. (*A*) Phase diagram of the cup dynamics. Averages of three independent runs with $r_{\mathrm{init}} = 1.5$ µm. (B-G) Time course of the active patch development in a quasi-3 dimensional space with z-axis symmetry. Parameters correspond to those in the order of Figure 2*A*, 2*C*, 2*F*, 2*D*, 2*E* and 2*G*.
